# Circulating *n*-3 fatty acids and *trans*-fatty acids, *PLA2G2A* gene variation and sudden cardiac arrest

**DOI:** 10.1017/jns.2016.2

**Published:** 2016-03-01

**Authors:** Rozenn N. Lemaitre, Traci M. Bartz, Irena B. King, Jennifer A. Brody, Barbara McKnight, Nona Sotoodehnia, Thomas D. Rea, Catherine O. Johnson, Dariush Mozaffarian, Stephanie Hesselson, Pui-Yan Kwok, David S. Siscovick

**Affiliations:** 1 Cardiovascular Health Research Unit, Department of Medicine, University of Washington, Seattle, WA, USA; 2 Cardiovascular Health Research Unit, Department of Biostatistics, University of Washington, Seattle, WA, USA; 3 Department of Internal Medicine, University of New Mexico, Albuquerque, NM, USA; 4 Friedman School of Nutrition Science and Policy, Tufts University, Boston, MA, USA; 5 Cardiovascular Research Institute and Institute for Human Genetics, University of California, San Francisco, CA, USA; 6 New York Academy of Medicine, New York, NY, USA

**Keywords:** Sudden cardiac arrest, Genetics, Fatty acids, Epidemiology, SCA, sudden cardiac arrest, sPLA2, secretory phospholipase A2

## Abstract

Whether genetic factors influence the associations of fatty acids with the risk of sudden cardiac arrest (SCA) is largely unknown. To investigate possible gene–fatty acid interactions on SCA risk, we used a case-only approach and measured fatty acids in erythrocyte samples from 1869 SCA cases in a population-based repository with genetic data. We selected 191 SNP in ENCODE-identified regulatory regions of fifty-five candidate genes in fatty acid metabolic pathways. Using linear regression and additive genetic models, we investigated the association of the selected SNP with erythrocyte levels of fatty acids, including DHA, EPA and *trans*-fatty acids among the SCA cases. The assumption of no association in non-cases was supported by analysis of publicly available datasets containing over 8000 samples. None of the SNP–fatty acid associations tested among the cases reached statistical significance after correction for multiple comparisons. One SNP, rs4654990 near *PLA2G2A*, with an allele frequency of 0·33, was nominally associated with lower levels of DHA and EPA and higher levels of *trans*-fatty acids. The strongest association was with DHA levels (exponentiated coefficient for one unit (1 % of total fatty acids), 0·90, 95 % CI 0·85, 0·97; *P* = 0·003), indicating that for subjects with a coded allele, the OR of SCA associated with one unit higher DHA is about 90 % what it is for subjects with one fewer coded allele. These findings suggest that the associations of circulating *n*-3 and *trans*-fatty acids with SCA risk may be more pronounced in carriers of the rs4654990 G allele.

Levels of circulating fatty acids are associated with risk of incident sudden cardiac arrest (SCA). In particular, higher levels of very long-chain *n*-3 fatty acids are associated with lower risk and higher levels of *trans*-fatty acids, especially *trans* isomers of linoleic acid (*trans*-18 : 2), are associated with higher risk of SCA^(^^1^^–^^5^^)^. In addition to circulating fatty acids and other risk factors, common genetic variation may also contribute to SCA risk^(^^6^^–^^11^^)^. In agreement with a possible involvement of fatty acid composition in SCA risk, we have provided suggestive evidence that genetic variation in *LPCAT1*, a gene involved in remodelling of phospholipid fatty acids, is associated with incident SCA^(^^12^^)^. Whether genetic factors also modify fatty acid associations with SCA is largely unknown.

To investigate possible gene–fatty acid interactions, we used a case-only approach. We measured fatty acids in erythrocyte samples from 1869 SCA patients, on whom we have also assessed genetic variation in fatty acid metabolic pathways^(^^12^^)^. For this investigation, we focused on genes in metabolic pathways downstream from PUFA, namely eicosanoid pathways, as well as genes involved in the use of fatty acids as an energy source. We hypothesised that variation in these genes would modify the association of circulating DHA, EPA and *trans*-fatty acids with incident SCA.

## Materials and methods

### Design

We investigated possible gene–fatty acid interactions on the risk of SCA using a case-only design. Under the assumption of no association in non-cases, coefficients for SNP–fatty acid associations among cases are estimates of coefficients for SNP–fatty acid interactions on the outcome of SCA^(^^13^^)^.

### Study population

Cases were selected from the Cardiac Arrest Blood Study Repository, a large population-based repository of data and specimens from adult out-of-hospital cardiac arrest patients who were attended by paramedics in Seattle and King county, Washington^(^^14^^)^. The study was restricted to 1869 SCA patients of European descent with cardiac arrest from 1989 to 2004, with initial rhythm of ventricular fibrillation or asystole, and with fatty acid and genetic data. The research was conducted according to the Declaration of Helsinki. The Human Subject Review Committee of the University of Washington approved the study and the collection of data and specimens in the Cardiac Arrest Blood Study Repository under a waiver of consent.

### Blood collection

Paramedics obtained blood specimens from cases in the field after all emergency medical care had been provided and the patient was either clinically stable or deceased, usually within 30 min of the cardiac arrest^(^^1^^)^. Blood was collected in tubes containing EDTA. Plasma, leucocytes and washed erythrocytes were stored at −80°C. DNA was extracted from leucocytes using standard phenol extraction procedures.

### Genotyping and SNP selection

Genotyping was performed on the Affymetrix Axiom panel in the laboratory of P.-Y. K. (Cardiovascular Research Institute, University of California, San Francisco, CA). Sample exclusion criteria were call rates <90 %, sex mismatches or non-European by principal component analysis. SNP exclusion criteria were call rate <97 %, discordant across duplicates or out of Hardy–Weinberg equilibrium (*P* < 10^−5^). A set of 522 985 autosomal SNP were imputed into the 566 European Ancestry haplotypes from the 1000G 2010-08 release using minimac^(^^15^^)^.

SNP for the study, from candidate gene regions ±50 kB, were extracted from the 1000G 2010-08 release. SNP with a minor allele frequency of <10 % or an imputation quality <0·8 were removed. The selected SNP were intersected with ENCODE regulatory regions from cardiac fibroblasts (HCF), atrial cardiac fibroblasts (HCFaa) or cardiac myocytes (HCM). We used the ENCODE annotation tracks DNAase I hypersensitivity sites, H3K4me3 Histone marks and ChipSeq CTCF sites. All tracks used the HotSpots algorithm for interval selection. Data were extracted from the UCSC Genome Browser (http://genome.ucsc.edu/ENCODE/). SNP falling in at least one ENCODE site were then linkage disequilibrium pruned in PLINK using the genotypes from the European samples of the 2012-03 1000G release and an *r*^2^ threshold of 0·5. A set of 191 SNP in potentially regulatory regions was included in the study. In addition, we examined a SNP in *PLA2G2A*, rs4654990, that is known to affect circulatory levels of the coded enzyme^(^^16^^–^^18^^)^.

### Fatty acid assays

Fatty acids were extracted using the method of Rose & Oklander^(^^19^^)^ and fatty acid methyl esters were prepared by direct transesterification using the method of Lepage & Roy^(^^20^^)^. The fatty acid methyl esters were separated by gas–liquid chromatography using a 100 m × 0·25 mm capillary silica column as described previously^(^^4^^)^. A total of forty-two fatty acids were assessed. Fatty acid levels are expressed as percentages of total fatty acids. *Trans*-isomers with eighteen carbons and one double bond (referred to as *trans*-18 : 1) are the sum of five isomers; *trans*-isomers of linoleic acid (*trans*-18 : 2) are the sum of three isomers.

### Statistical methods

Analyses were carried out using Stata 13·0 (StataCorp). Associations of SNP with fatty acid levels were assessed using linear regression. We used additive genetic models adjusted for age, sex and categories of calendar year. Separate models were used for each SNP and each fatty acid. We used a threshold *P* value for significance of 0·00026 (0·05/191 tested SNP) to correct for multiple comparisons. In sensitivity analyses, we repeated the analyses restricted to SCA patients with initial rhythm of ventricular fibrillation.

Case-only analyses rely on the assumption of no associations in non-cases. To examine this assumption, we looked at the associations of rs4654990 with fatty acids^(^^21^^,^^22^^)^ *in situ* in publicly available results of genome-wide association studies of fatty acids from the Cohorts for Heart and Aging Research in Genomic Epidemiology (CHARGE) Consortium (http://www.chargeconsortium.com/main/results).

To obtain estimates of the main effect of fatty acids on SCA risk, we used data from a case–control study that included a subset of the repository cases, without prior diagnosed heart disease, and individually matched controls^(^^4^^)^. We used conditional logistic regression to account for matching factors (age, sex and calendar year) and further adjusted for SCA risk factors (smoking, diabetes, hypertension, education, weight, height, leisure time physical activity and fat intake). These analyses, which have been reported earlier^(^^1^^,^^4^^)^, were repeated to obtain estimates of the association of one unit higher fatty acid (1 % of total fatty acids) with SCA risk. We did not attempt replication of the case-only findings with the case–control data set because power to detect an interaction of similar magnitude as that of rs4654990 with DHA was only 20 % with the case–control data.

## Results

Demographics of the 1869 SCA cases included in the study, and erythrocyte membrane levels of DHA, EPA and *trans*-fatty acids are shown in Table 1. Levels of DHA correlated with EPA (*r* 0·57) and levels of *trans*-18 : 1 with *trans*-18 : 2 (*r* 0·47); correlations between DHA/EPA and the *trans*-fatty acids were negative, ranging from −0·10 to −0·28 (Supplementary Table S1).

**Table 1.** Characteristics of 1869 sudden cardiac arrest cases in the study (Mean values and standard deviations, or percentages)

Characteristic	Mean	sd
Age (years)	67·99	13·88
Men (%)	76·19	
Initial rhythm of ventricular fibrillation (%)	70·52	
History of coronary artery disease* (%)	41·21	
Erythrocyte DHA (% of total fatty acids)	3·37	0·98
Erythrocyte EPA (% of total fatty acids)	0·46	0·22
Erythrocyte *trans*-18 : 1 (% of total fatty acids)	1·74	0·58
Erythrocyte *trans*-18 : 2 (% of total fatty acids)	0·21	0·058

* Among 1206 cases with prior hospital records.

We investigated possible SNP–fatty acid interactions on the risk of SCA in case-only analyses. In the SCA cases, we examined the associations of 191 SNP in regulatory regions of fifty-five candidate genes (Supplementary Table S2) with levels of EPA, DHA and *trans*-fatty acids. At the preset threshold level of *P* = 0·00026, no SNP–fatty acid association reached statistical significance (Supplementary Tables S3–S6). The SNP that was most associated with DHA among cases (*P* = 0·003) was also nominally associated with the other three fatty acids (Table 2). The G allele of rs4654990, near *PLA2G2A*, with 0·33 allele frequency, was nominally inversely associated with DHA and EPA, and positively associated with *trans*-18 : 1 and *trans*-18 : 2 fatty acids. The strongest association was with DHA levels (exponentiated coefficient for one unit (1 % of total fatty acids): 0·905; 95 % CI 0·85, 0·97), indicating that for subjects with a coded allele, the OR associated with one unit higher DHA is about 90 % what it is for subjects with one fewer coded allele.

**Table 2.** Association of the G allele of rs4654990 with erythrocyte fatty acids in 1869 sudden cardiac arrest cases (Coefficients with their standard errors)

Fatty acid	Coefficient*	se	Exponentiated coefficient	*P*
DHA	−0·099	0·034	0·905	0·003
EPA	−0·020	0·008	0·981	0·013
*Trans*-18 : 1	0·055	0·019	1·056	0·005
*Trans*-18 : 2	0·004	0·002	1·004	0·045

* Coefficient for an increase in one unit of fatty acid (1 % of total fatty acids).

Among the three *trans*-18 : 2 isomers, both the *cis, trans*- and the *trans, cis*-18 : 2 were associated with rs4654990 (regression coefficient: 0·002 (se 0·001) for both isomers; *P* = 0·048 and 0·041, respectively). In sensitivity analyses restricted to cases found in ventricular fibrillation, we observed similar associations of rs4654990 with fatty acids (Supplementary Table S7).

Coefficients for SNP–fatty acid associations among SCA cases are estimates of coefficients for SNP–fatty acid interactions on the outcome of SCA under the assumption of no association in non-cases^(^^13^^)^. We examined this assumption using public data from the CHARGE Consortium^(^^21^^,^^22^^)^. In meta-analyses including over 8000 study samples, none of the fatty acids was associated with rs4654990 (*P* > 0·4; Table 3).

**Table 3.** Associations of the G allele of rs4654990 with plasma phospholipid levels of fatty acids in meta-analysis results from the Cohorts for Heart and Aging Research in Genomic Epidemiology (CHARGE) Consortium* (Coefficients with their standard errors, and 95 % confidence intervals)

Fatty acid	Coefficient†	se	95 % CI	*P*
DHA‡	0·0029	0·0152	−0·0008, 0·033	0·85
EPA‡	−0·0005	0·0054	−0·011, 0·010	0·93
*Trans*-18 : 1§	−0·0060	0·0102	−0·026, 0·014	0·56
*Trans*-18 : 2	NA			
*Trans*-18 : 2ct§	−0·0004	0·0004	−0·0012, 0·0004	0·43
*Trans*-18 : 2tc§	−0·0005	0·0009	−0·0023, 0·0013	0·59

NA, not available.

* Results from http://www.chargeconsortium.com/main/results

† Coefficient for an increase in one unit of fatty acid (1 % of total fatty acids).

‡ Results of associations in 8866 samples^(^^22^^)^.

§ Results of associations in 8013 samples^(^^21^^)^.

Case-only analyses provide estimates of interactions, but not of main effects. A previous report that used the same case population together with population controls showed no main effect of genetic variation in *PLA2G2A* on SCA risk^(^^12^^)^. However, we have reported the main effects of fatty acids on SCA risk using a subset of the SCA cases included here and randomly selected, individually matched controls. In particular, we reported associations of higher levels of erythrocyte DHA and EPA with lower risk, and higher levels of *trans*-fatty acids with higher risk of incident SCA^(^^1^^,^^4^^)^. Using the data from that case–control study^(^^4^^)^, the estimated regression coefficients for the association of an increase of one unit in fatty acid (1 % of total fatty acids) were −0·32 (se 0·09) for DHA, −0·90 (se 0·41) for EPA, 0·38 (se 0·19) for *trans*-18 : 1 and 10·32 (se 2·58) for *trans*-18 : 2. These regression coefficients together with the coefficients from case-only analyses (Table 2), which approximate interaction coefficients, suggest that the associations of fatty acids with SCA risk are more pronounced in the presence of the G allele of rs4654990.

## Discussion

In this large case-only study of 1869 SCA cases from the community, we found no evidence for SNP x fatty acid interactions at our preset threshold of statistical significance (*P* = 0·00026). One SNP near *PLA2G2A* was nominally associated with higher EPA and DHA levels and lower *trans*-fatty acid levels among the SCA cases, but not in a large population sample. If replicated, this finding suggests a possible interaction such that *n*-3 and *trans*-fatty acids associations with SCA risk may be more pronounced in the presence of the G allele of rs4654990.

The gene *PLA2G2A* codes for a secretory phospholipase A2 (sPLA2, type IIA). Circulating levels of sPLA2-IIA are associated with incident coronary events and mortality^(^^23^^–^^26^^)^. Whether sPLA2 levels are also associated with SCA risk is not known. Phospholipases A2 release fatty acids from phospholipids, clipping the fatty acid in the *sn*-2 position where PUFA, including DHA and EPA, are located. The released fatty acid may influence the risk of arrhythmia. For example, in dogs with experimental ischaemia, increased levels of circulating un-esterified DHA protect from ventricular fibrillation^(^^27^^)^. In addition, PUFA liberated by sPLA2-IIA may be metabolised into eicosanoids^(^^28^^)^, and prostaglandins derived from EPA and DHA show fewer arrhythmic properties than those from arachidonic acid in cultured myocytes^(^^29^^)^. Whether the release of DHA or EPA explains an interaction with rs4654990 and enhances these fatty acids’ association with lower SCA risk is not known.

Study strengths include the hypothesis-based investigation, the large number of well-phenotyped incident SCA cases from the community, the use of objective fatty acid biomarkers, and the verification of the case-only assumption in a large dataset from population studies. Several limitations also need to be considered. Blood was collected after cardiac arrest, on average 30 min later; however, *n*-3 and *trans*-fatty acid associations with SCA in the study^(^^1^^,^^4^^)^ have been replicated in prospective studies^(^^2^^,^^3^^)^, suggesting minimal short-term impact on erythrocyte fatty acid composition. We measured fatty acids in different compartments among SCA cases (erythrocyte membranes) and the population sample (plasma phospholipids) used to verify the assumption of no SNP association in non-cases; however, we have shown similar main effects of genetic variants on plasma phospholipid and erythrocyte *n*-3 and *trans*-fatty acids^(^^21^^,^^30^^)^. Because case-only studies rely on the assumption of no association in non-cases, we were not able to study interactions with other potentially interesting candidate genes, such as the fatty acid desaturase (*FADS*) genes, that are associated with fatty acids in non-cases^(^^22^^)^. While we selected SNP from regulatory regions, the influence of genetic variation at rs4654990 on *PLA2G2A* transcription and sPLA2-IIA mass or activity is not known. Another SNP in *PLA2G2A* that is associated with circulating sPLA2-IIA mass did not show any association with the fatty acids, however, the SNP association with sPLA2-IIA activity appears low and inconsistent^(^^16^^)^. Due to multiple comparisons, the associations of rs4654990 could be due to chance. In spite of the large number of SCA cases, the study had limited power to detect associations.

In summary, we observed a nominal interaction of a SNP in a phospholipase A2 gene with erythrocyte membrane *n*-3 and *trans*-fatty acids, such that the associations of SCA risk with the fatty acids, especially DHA, appear more pronounced in the presence of the G allele of rs4654990. This finding needs to be confirmed.

## Supplementary material

For supplementary material accompanying this paper visit http://dx.doi.org/10.1017/jns.2016.2. click here to view supplementary material
