# Supplementary material for: Circulating n-3 fatty acids and trans-fatty acids, PLA2G2A gene variation and sudden cardiac arrest
Source: J Nutr Sci. 2016 Mar 1;5:e12. doi: 10.1017/jns.2016.2 (PMC4791519; doi:10.1017/jns.2016.2)
Supplement: Supplementary file 1 [file S2048679016000021sup.zip › S2048679016000021sup001.docx]

Supplementary table 1. Correlations between the 4 fatty acids in 1869 sudden cardiac arrest patients in GxE analyses

| **Fatty acid** | DHA | EPA | Trans-18:1 |
| --- | --- | --- | --- |
| DHA | 1 |  |  |
| EPA | 0.57 | 1 |  |
| Trans-18:1 | -0.17 | -0.28 | 1 |
| Trans-18:2 | -0.14 | -0.10 | 0.47 |
