# Supplementary material for: Circulating n-3 fatty acids and trans-fatty acids, PLA2G2A gene variation and sudden cardiac arrest
Source: J Nutr Sci. 2016 Mar 1;5:e12. doi: 10.1017/jns.2016.2 (PMC4791519; doi:10.1017/jns.2016.2)
Supplement: Supplementary file 1 [file S2048679016000021sup.zip › S2048679016000021sup002.docx]

Supplementary table 2. List of 55 selected candidate genes from fatty acid metabolism in GxE analyses.

| Gene symbol | Gene name | # SNPs |
| --- | --- | --- |
| ACACA | Acetyl-CoA carboxylase, subunit A | 6 |
| ACACB | Acetyl-CoA carboxylase, subunit B | 6 |
| ACADM | Acyl-CoA dehydrogenase, medium chain | 3 |
| ACADVL | Acyl-CoA dehydrogenase, very long chain | 2 |
| ACAT1 | Acetyl-CoA acetyl transferase | 4 |
| AKR1C3 | Prostaglandin F synthase | 7 |
| ALOX12 | Arachidonate 12-lipoxygenase | 3 |
| ALOX15 | Arachidonate 15-lipoxygenase | 1 |
| ALOX5 | Arachidonate 5-lipoxygenase | 3 |
| ALOX5AP | Arachidonate 5-lipoxygenase activating protein | 9 |
| CBR1 | PGE 9-reductase | 6 |
| CPT2 | Carnitine palmitoyltransferase 2 | 4 |
| CYP2J2 | Arachidonic acid epoxygenase | 4 |
| DECR1 | Dienoyl-CoA reductase 1 | 1 |
| DHRS4 | Dehydrogenase/reductase, SDR family | 1 |
| EPHX2 | Epoxide hydrolase 2 | 5 |
| GGT1 | Gamma-glutamyl transferase 1 | 1 |
| GGT5 | Gamma-glutamyl transferase 5 | 1 |
| GGT7 | Gamma-glutamyl transferase 7 | 2 |
| GPX3 | Glutathione peroxidase 3 | 7 |
| GPX7 | Glutathione peroxidase 7 | 4 |
| HADH | Hydroxyacyl-CoA dehydrogenase | 2 |
| HADHA | Hydroxyacyl-CoA dehydrogenase/3-ketoacyl-CoA thiolase/enoyl-CoA hydratase, alpha | 1 |
| HADHB | Hydroxyacyl-CoA dehydrogenase/3-ketoacyl-CoA thiolase/enoyl-CoA hydratase, beta | 2 |
| HPGD | Hydroxy prostaglandin dehydrogenase | 6 |
| LPL | Lipoprotein lipase | 3 |
| LTA4H | Leukotriene A4 hydrolase | 9 |
| LTC4S | Leukotriene-C4 synthase | 2 |
| MLYCD | Malonyl-CoA decarboxylase | 1 |
| PLA2G12A | Phospholipase A2, XIIA | 2 |
| PLA2G12B | Phospholipase A2, XIIB | 1 |
| PLA2G2A | Phospholipase A2, IIA | 6 |
| PLA2G4A | Phospholipase A2, IVA | 7 |
| PLA2G5 | Phospholipase A2, V | 5 |
| PLA2G7 | Phospholipase A2, VII | 2 |
| PPARGC1A | Peroxisome proliferators-activated receptor γ co-activator 1 | 8 |
| PRKAA2 | AMP-activated protein kinase α2 subunit | 5 |
| PRKAB1 | AMP-activated protein kinase ß1 subunit | 2 |
| PRKAB2 | AMP-activated protein kinase ß2 subunit | 2 |
| PRKAG1 | AMP-activated protein kinase γ1 subunit | 4 |
| PRKAG3 | AMP-activated protein kinase γ3 subunit | 2 |
| PTGDS | Prostaglandin D synthase | 1 |
| PTGER3 | Prostaglandin E receptor 3 | 6 |
| PTGES | Prostaglandin E synthase | 3 |
| PTGES2 | Prostaglandin E synthase 2 | 1 |
| PTGFRN | Prostaglandin F2 receptor negative regulator | 5 |
| PTGIS | Prostacyclin synthase | 3 |
| PTGS1 | Cyclooxygenase 1 | 3 |
| PTGS2 | Cyclooxygenase 2 | 5 |
| SLC25A20 | Carnitine acylcarnitine translocase | 1 |
| TBXAS1 | Thromboxane A synthase | 12 |
