# Supplementary material for: Circulating n-3 fatty acids and trans-fatty acids, PLA2G2A gene variation and sudden cardiac arrest
Source: J Nutr Sci. 2016 Mar 1;5:e12. doi: 10.1017/jns.2016.2 (PMC4791519; doi:10.1017/jns.2016.2)
Supplement: Supplementary file 1 [file S2048679016000021sup.zip › S2048679016000021sup003.docx]

Supplementary table 3: Comprehensive results for DHA

| Position | Marker | Chrom | Coded allele | gene | Beta coef | SE | p-value |
| --- | --- | --- | --- | --- | --- | --- | --- |
| 4582183 | rs55682338 | 17 | G | ALOX15 | 0.04004 | 0.038682 | 0.300624 |
| 5051532 | rs1937863 | 10 | G | AKR1C3 | -0.063247 | 0.042189 | 0.133835 |
| 5060658 | rs1937887 | 10 | T | AKR1C3 | 0.022575 | 0.052382 | 0.666494 |
| 5085931 | rs56114059 | 10 | G | AKR1C3 | -0.02871 | 0.046183 | 0.534165 |
| 5113711 | rs4881394 | 10 | T | AKR1C3 | -0.007436 | 0.034959 | 0.831556 |
| 5114183 | rs34477787 | 10 | C | AKR1C3 | -0.001857 | 0.044303 | 0.966572 |
| 5134511 | rs1937847 | 10 | A | AKR1C3 | 0.022103 | 0.041986 | 0.598581 |
| 5138607 | rs7741 | 10 | G | AKR1C3 | 0.015845 | 0.032627 | 0.62722 |
| 6899629 | rs3218667 | 17 | C | ALOX12 | -0.0309 | 0.03295 | 0.348344 |
| 6915401 | rs2271316 | 17 | C | ALOX12 | -0.002168 | 0.035003 | 0.950622 |
| 6963609 | rs11654772 | 17 | C | ALOX12 | -0.075799 | 0.037634 | 0.043999 |
| 7089652 | rs314256 | 17 | C | ACADVL | 0.020081 | 0.035362 | 0.570128 |
| 7154582 | rs3744399 | 17 | T | ACADVL | -0.019859 | 0.045424 | 0.661969 |
| 19748603 | rs7844579 | 8 | T | LPL | 0.05114 | 0.031632 | 0.105946 |
| 19797916 | rs3779787 | 8 | G | LPL | -0.028461 | 0.044765 | 0.524911 |
| 19869676 | rs1919484 | 8 | G | LPL | -0.009855 | 0.036768 | 0.788683 |
| 20273680 | rs4654990 | 1 | G | PLA2G2A | -0.099045 | 0.033815 | 0.0034 |
| 20275868 | rs12083280 | 1 | G | PLA2G2A | 0.056488 | 0.040444 | 0.162511 |
| 20301781 | rs3767221 | 1 | A | PLA2G2A | -0.003493 | 0.033619 | 0.917243 |
| 20306146 | rs11573156 | 1 | C | PLA2G2A | -0.050066 | 0.041686 | 0.229737 |
| 20334912 | rs10916689 | 1 | G | PLA2G2A | -0.045848 | 0.040534 | 0.258018 |
| 20335423 | rs4233290 | 1 | G | PLA2G2A | 0.006082 | 0.044825 | 0.892065 |
| 20357803 | rs818675 | 1 | T | PLA2G5 | -0.047107 | 0.03258 | 0.148205 |
| 20414239 | rs11573272 | 1 | A | PLA2G5 | 0.021387 | 0.032714 | 0.513273 |
| 20420414 | rs61770054 | 1 | A | PLA2G5 | -0.029668 | 0.044312 | 0.503157 |
| 20437563 | rs7518058 | 1 | T | PLA2G5 | -0.014185 | 0.034455 | 0.680567 |
| 20451812 | rs636584 | 1 | C | PLA2G5 | -0.003485 | 0.031862 | 0.912899 |
| 23790132 | rs2970884 | 4 | C | PPARGC1A | -0.034657 | 0.035678 | 0.331352 |
| 23858960 | rs11941854 | 4 | T | PPARGC1A | 0.057664 | 0.042021 | 0.16998 |
| 23859103 | rs34478957 | 4 | C | PPARGC1A | -0.028297 | 0.032821 | 0.388599 |
| 23886131 | rs35121232 | 4 | C | PPARGC1A | 0.033198 | 0.05157 | 0.519735 |
| 23886323 | rs2946385 | 4 | G | PPARGC1A | -0.033099 | 0.030992 | 0.285513 |
| 23889781 | rs2970873 | 4 | A | PPARGC1A | -0.112261 | 0.044447 | 0.011546 |
| 23934551 | rs12642645 | 4 | A | PPARGC1A | 0.024944 | 0.031885 | 0.434031 |
| 23934688 | rs6851904 | 4 | C | PPARGC1A | -0.009885 | 0.036046 | 0.783907 |
| 24380071 | rs17794681 | 14 | C | DHRS4 | -0.000128 | 0.038681 | 0.997365 |
| 24610727 | rs7284722 | 22 | G | GGT5 | -0.050484 | 0.043567 | 0.246543 |
| 24946618 | rs9624495 | 22 | G | GGT1 | 0.019351 | 0.050317 | 0.700553 |
| 26465848 | rs6752314 | 2 | C | HADHB | -0.014081 | 0.044262 | 0.750391 |
| 26491392 | rs10445947 | 2 | A | HADHB | -0.034769 | 0.034732 | 0.316804 |
| 26549127 | rs11689086 | 2 | C | HADHB | -0.061019 | 0.033319 | 0.06705 |
| 27323607 | rs2322581 | 8 | G | EPHX2 | -0.05063 | 0.044497 | 0.255193 |
| 27381996 | rs721619 | 8 | C | EPHX2 | -0.051947 | 0.03286 | 0.113909 |
| 27402494 | rs4149259 | 8 | C | EPHX2 | 0.013197 | 0.0442 | 0.765268 |
| 27405576 | rs2640727 | 8 | C | EPHX2 | -0.003273 | 0.0355 | 0.926549 |
| 27450251 | rs10111053 | 8 | G | EPHX2 | 0.01891 | 0.03845 | 0.622859 |
| 31247510 | rs61947822 | 13 | T | ALOX5AP | -0.077247 | 0.05261 | 0.14202 |
| 31256268 | rs9508815 | 13 | C | ALOX5AP | 0.028805 | 0.034661 | 0.405945 |
| 31271809 | rs3000632 | 13 | A | ALOX5AP | 0.009015 | 0.032819 | 0.783563 |
| 31296538 | rs9743182 | 13 | T | ALOX5AP | -0.137023 | 0.048777 | 0.004967 |
| 31299553 | rs17222814 | 13 | G | ALOX5AP | 0.001145 | 0.053452 | 0.982917 |
| 31317878 | rs4254165 | 13 | A | ALOX5AP | -0.013789 | 0.034668 | 0.690817 |
| 31318020 | rs4360791 | 13 | G | ALOX5AP | -0.006856 | 0.03224 | 0.831588 |
| 31366810 | rs4238140 | 13 | A | ALOX5AP | -0.007063 | 0.042815 | 0.868973 |
| 31370547 | rs9315067 | 13 | G | ALOX5AP | 0.01711 | 0.036599 | 0.640148 |
| 33413176 | rs7262274 | 20 | G | GGT7 | -0.037839 | 0.032723 | 0.24754 |
| 33460366 | rs11697978 | 20 | G | GGT7 | -0.003988 | 0.040967 | 0.922457 |
| 35475913 | rs7215365 | 17 | C | ACACA | -0.023454 | 0.042423 | 0.580355 |
| 35476626 | rs17573357 | 17 | A | ACACA | 0.041907 | 0.047166 | 0.374278 |
| 35547802 | rs11653093 | 17 | C | ACACA | 0.031397 | 0.031457 | 0.318235 |
| 35754910 | rs35707017 | 17 | C | ACACA | -0.034799 | 0.038407 | 0.364917 |
| 35760940 | rs1102920 | 17 | A | ACACA | -0.080436 | 0.043285 | 0.063126 |
| 35767603 | rs829165 | 17 | T | ACACA | 0.051326 | 0.044232 | 0.245893 |
| 37395556 | rs4817761 | 21 | C | CBR1 | 0.054898 | 0.033491 | 0.101173 |
| 37443480 | rs1005696 | 21 | T | CBR1 | 0.008468 | 0.033207 | 0.798712 |
| 37443671 | rs2156406 | 21 | A | CBR1 | -0.029877 | 0.050892 | 0.557158 |
| 37443893 | rs3787728 | 21 | T | CBR1 | 0.022882 | 0.035426 | 0.518342 |
| 37494978 | rs4816519 | 21 | A | CBR1 | -0.001614 | 0.04776 | 0.973045 |
| 37495374 | rs7283730 | 21 | G | CBR1 | 0.04516 | 0.033109 | 0.172573 |
| 45869713 | rs4987106 | 10 | C | ALOX5 | -0.002092 | 0.047257 | 0.964685 |
| 45915941 | rs2099171 | 10 | C | ALOX5 | -0.048976 | 0.036501 | 0.179668 |
| 45938239 | rs2242332 | 10 | T | ALOX5 | 0.027569 | 0.035152 | 0.432864 |
| 46702601 | rs6935058 | 6 | A | PLA2G7 | -0.061357 | 0.045695 | 0.17935 |
| 46703319 | rs9395208 | 6 | G | PLA2G7 | -0.047127 | 0.039256 | 0.229935 |
| 48125015 | rs13039739 | 20 | G | PTGIS | -0.043357 | 0.040504 | 0.284423 |
| 48196962 | rs538748 | 20 | G | PTGIS | 0.002813 | 0.035058 | 0.936049 |
| 48210099 | rs508864 | 20 | C | PTGIS | -0.075908 | 0.039035 | 0.051824 |
| 48893780 | rs9840684 | 3 | T | SLC25A20 | -0.013399 | 0.035841 | 0.708509 |
| 49351654 | rs833827 | 12 | A | PRKAG1 | 0.012048 | 0.034491 | 0.726856 |
| 49389320 | rs1054442 | 12 | A | PRKAG1 | 0.051428 | 0.03494 | 0.141043 |
| 49451459 | rs833836 | 12 | C | PRKAG1 | 0.022221 | 0.043397 | 0.608627 |
| 49455006 | rs11168838 | 12 | A | PRKAG1 | 0.054722 | 0.036813 | 0.137146 |
| 53037658 | rs2452763 | 1 | G | GPX7 | -0.013072 | 0.049116 | 0.790135 |
| 53068093 | rs11205977 | 1 | G | GPX7 | 0.117118 | 0.05017 | 0.019574 |
| 53068430 | rs12089784 | 1 | G | GPX7 | 0.030499 | 0.031679 | 0.335669 |
| 53098558 | rs12728876 | 1 | A | GPX7 | -0.004226 | 0.045074 | 0.925311 |
| 53613018 | rs1679936 | 1 | G | CPT2 | 0.019468 | 0.032584 | 0.550198 |
| 53613907 | rs6696614 | 1 | A | CPT2 | -0.021048 | 0.034679 | 0.543901 |
| 53663128 | rs3766760 | 1 | G | CPT2 | 0.00981 | 0.033139 | 0.76722 |
| 53686383 | rs1679913 | 1 | C | CPT2 | -0.037183 | 0.034314 | 0.278537 |
| 57072543 | rs2796540 | 1 | G | PRKAA2 | 0.016575 | 0.031629 | 0.600252 |
| 57092231 | rs7542282 | 1 | A | PRKAA2 | -0.03331 | 0.051044 | 0.514037 |
| 57202872 | rs6656021 | 1 | A | PRKAA2 | 0.041414 | 0.041518 | 0.31852 |
| 57223201 | rs10489620 | 1 | C | PRKAA2 | 0.015383 | 0.031912 | 0.629779 |
| 57223222 | rs857105 | 1 | G | PRKAA2 | -0.025582 | 0.034126 | 0.453468 |
| 60392271 | rs11572191 | 1 | G | CYP2J2 | 0.079111 | 0.051416 | 0.123893 |
| 60413890 | rs7518613 | 1 | T | CYP2J2 | 0.080742 | 0.051969 | 0.120271 |
| 60413925 | rs11207546 | 1 | C | CYP2J2 | -0.008403 | 0.034619 | 0.808208 |
| 60436924 | rs877494 | 1 | C | CYP2J2 | 0.019144 | 0.033707 | 0.570066 |
| 71284954 | rs1327453 | 1 | G | PTGER3 | -0.052667 | 0.03311 | 0.111682 |
| 71398588 | rs12067140 | 1 | A | PTGER3 | 0.02643 | 0.049735 | 0.595128 |
| 71432984 | rs626398 | 1 | A | PTGER3 | 0.069039 | 0.044157 | 0.117941 |
| 71444355 | rs601934 | 1 | T | PTGER3 | -0.019388 | 0.032527 | 0.551134 |
| 71549987 | rs11808123 | 1 | A | PTGER3 | -0.017634 | 0.050951 | 0.729272 |
| 71556167 | rs7531139 | 1 | A | PTGER3 | -0.005528 | 0.0405 | 0.891433 |
| 74741181 | rs7893781 | 10 | T | PLA2G12B | 0.006585 | 0.032645 | 0.840137 |
| 76191744 | rs11161465 | 1 | T | ACADM | -0.005815 | 0.035205 | 0.868799 |
| 76252335 | rs1146635 | 1 | A | ACADM | -0.066029 | 0.032822 | 0.044248 |
| 76255228 | rs11161620 | 1 | G | ACADM | 0.069068 | 0.036269 | 0.056867 |
| 83912536 | rs12716744 | 16 | A | MLYCD | 0.001649 | 0.035014 | 0.962434 |
| 90996258 | rs1805844 | 8 | A | DECR1 | 0.012568 | 0.034364 | 0.714558 |
| 96357235 | rs7486464 | 12 | A | LTA4H | 0.041898 | 0.033184 | 0.206736 |
| 96374750 | rs2270318 | 12 | G | LTA4H | -0.047152 | 0.032398 | 0.145557 |
| 96408976 | rs2072512 | 12 | T | LTA4H | 0.013823 | 0.031921 | 0.664983 |
| 96437903 | rs2660842 | 12 | A | LTA4H | -0.062301 | 0.036922 | 0.091532 |
| 96437926 | rs2660843 | 12 | A | LTA4H | -0.027674 | 0.040036 | 0.489426 |
| 96451981 | rs4762661 | 12 | T | LTA4H | -0.084779 | 0.045312 | 0.061344 |
| 96455871 | rs2660885 | 12 | C | LTA4H | 0.004456 | 0.040451 | 0.91228 |
| 96464620 | rs7306046 | 12 | A | LTA4H | -0.089655 | 0.040038 | 0.02514 |
| 96485986 | rs58006709 | 12 | C | LTA4H | 0.022091 | 0.045375 | 0.626363 |
| 107947457 | rs7120711 | 11 | G | ACAT1 | 0.027778 | 0.03851 | 0.470709 |
| 107986691 | rs11601596 | 11 | T | ACAT1 | 0.026982 | 0.037857 | 0.476006 |
| 107992312 | rs3741054 | 11 | C | ACAT1 | -0.04039 | 0.041517 | 0.330632 |
| 108013093 | rs10890818 | 11 | A | ACAT1 | -0.011607 | 0.044927 | 0.796142 |
| 108912668 | rs6533329 | 4 | A | HADH | 0.054604 | 0.047207 | 0.247399 |
| 108931551 | rs141066 | 4 | A | HADH | 0.011703 | 0.03603 | 0.745314 |
| 109535809 | rs1018782 | 12 | A | ACACB | 0.028871 | 0.044851 | 0.519765 |
| 109549579 | rs7978946 | 12 | C | ACACB | -0.008696 | 0.040406 | 0.829595 |
| 109592483 | rs11610260 | 12 | T | ACACB | -0.002223 | 0.039475 | 0.955089 |
| 109693348 | rs55960723 | 12 | G | ACACB | -0.019013 | 0.054587 | 0.727604 |
| 109740442 | rs11066182 | 12 | G | ACACB | -0.035524 | 0.035181 | 0.31262 |
| 109740593 | rs11831762 | 12 | C | ACACB | 0.051691 | 0.051131 | 0.312033 |
| 110621820 | rs5030539 | 4 | A | PLA2G12A | -0.023606 | 0.032739 | 0.470881 |
| 110651146 | rs11555260 | 4 | C | PLA2G12A | 0.030044 | 0.033127 | 0.364439 |
| 117421595 | rs11807991 | 1 | G | PTGFRN | 0.104798 | 0.039959 | 0.008725 |
| 117487884 | rs10923173 | 1 | G | PTGFRN | -0.020358 | 0.033702 | 0.54581 |
| 117514334 | rs6687760 | 1 | A | PTGFRN | -0.026233 | 0.045157 | 0.561289 |
| 117540386 | rs2057592 | 1 | A | PTGFRN | -0.048918 | 0.034854 | 0.160465 |
| 117550327 | rs2806873 | 1 | C | PTGFRN | 0.003144 | 0.040186 | 0.937647 |
| 120097006 | rs7967558 | 12 | T | PRKAB1 | 0.003808 | 0.033775 | 0.910226 |
| 120105707 | rs6490265 | 12 | T | PRKAB1 | 0.027343 | 0.042946 | 0.524327 |
| 125139340 | rs35119072 | 9 | T | PTGS1 | -0.053904 | 0.045457 | 0.235696 |
| 125164936 | rs10513402 | 9 | T | PTGS1 | -0.052491 | 0.053872 | 0.329873 |
| 125180343 | rs2778618 | 9 | A | PTGS1 | -0.019215 | 0.045032 | 0.669594 |
| 130850583 | rs10987870 | 9 | T | PTGES2 | 0.003888 | 0.048029 | 0.935481 |
| 132523860 | rs10118377 | 9 | A | PTGES | -0.029616 | 0.035406 | 0.402881 |
| 132538919 | rs1017509 | 9 | T | PTGES | 0.036264 | 0.046157 | 0.432071 |
| 132552936 | rs11793199 | 9 | T | PTGES | -0.047551 | 0.037135 | 0.200365 |
| 139463264 | rs1011018 | 7 | G | TBXAS1 | 0.045142 | 0.042794 | 0.291493 |
| 139523858 | rs1990354 | 7 | A | TBXAS1 | 0.092839 | 0.032262 | 0.004006 |
| 139553641 | rs41706 | 7 | T | TBXAS1 | -0.084235 | 0.035725 | 0.01838 |
| 139596859 | rs2267691 | 7 | C | TBXAS1 | -0.06295 | 0.049478 | 0.203276 |
| 139609317 | rs1978180 | 7 | C | TBXAS1 | 0.09651 | 0.047319 | 0.041395 |
| 139618001 | rs2299891 | 7 | T | TBXAS1 | -0.04914 | 0.034581 | 0.155313 |
| 139640896 | rs4726473 | 7 | T | TBXAS1 | 0.075225 | 0.036157 | 0.037479 |
| 139687783 | rs2267703 | 7 | G | TBXAS1 | 4.00E-04 | 0.033648 | 0.990524 |
| 139695316 | rs2284212 | 7 | G | TBXAS1 | 0.017455 | 0.035353 | 0.621484 |
| 139741766 | rs10267006 | 7 | C | TBXAS1 | -0.005117 | 0.041137 | 0.901003 |
| 139761463 | rs7794528 | 7 | C | TBXAS1 | 0.022799 | 0.037612 | 0.544407 |
| 139763419 | rs6943771 | 7 | C | TBXAS1 | -0.112362 | 0.050599 | 0.026374 |
| 139822542 | rs908831 | 9 | C | PTGDS | 0.01727 | 0.031761 | 0.586622 |
| 146610792 | rs4397700 | 1 | C | PRKAB2 | -0.030122 | 0.051039 | 0.555068 |
| 146641686 | rs3766522 | 1 | A | PRKAB2 | 0.062698 | 0.036472 | 0.085602 |
| 150369314 | rs57652693 | 5 | G | GPX3 | 0.021033 | 0.044277 | 0.634762 |
| 150384959 | rs2054440 | 5 | A | GPX3 | -0.009534 | 0.031025 | 0.758608 |
| 150400587 | rs870407 | 5 | A | GPX3 | 0.017022 | 0.047248 | 0.718645 |
| 150404311 | rs8177433 | 5 | C | GPX3 | -0.001038 | 0.041794 | 0.980177 |
| 150428584 | rs4958878 | 5 | A | GPX3 | -0.006876 | 0.052135 | 0.895076 |
| 150428871 | rs6862024 | 5 | G | GPX3 | -0.010156 | 0.03419 | 0.76643 |
| 150455672 | rs3792784 | 5 | A | GPX3 | 0.0351 | 0.051333 | 0.494116 |
| 175411953 | rs9312555 | 4 | A | HPGD | 0.014324 | 0.044902 | 0.749728 |
| 175442822 | rs1365613 | 4 | T | HPGD | -0.050277 | 0.042591 | 0.237826 |
| 175444281 | rs17553108 | 4 | G | HPGD | 0.031638 | 0.047619 | 0.506435 |
| 175463488 | rs2256673 | 4 | T | HPGD | -0.030122 | 0.043107 | 0.484691 |
| 175463555 | rs2256669 | 4 | C | HPGD | -0.064764 | 0.039052 | 0.097236 |
| 175475048 | rs17060632 | 4 | A | HPGD | 0.04943 | 0.045912 | 0.281648 |
| 179177116 | rs55827522 | 5 | C | LTC4S | -0.014585 | 0.035208 | 0.678682 |
| 179250392 | rs10464093 | 5 | G | LTC4S | -0.009974 | 0.033775 | 0.767763 |
| 186612725 | rs10911899 | 1 | T | PTGS2 | -0.00239 | 0.050485 | 0.962234 |
| 186648197 | rs5277 | 1 | C | PTGS2 | -0.013865 | 0.042263 | 0.742861 |
| 186649221 | rs2745557 | 1 | A | PTGS2 | -0.039091 | 0.043201 | 0.365534 |
| 186650751 | rs689466 | 1 | T | PTGS2 | 0.01156 | 0.042719 | 0.786701 |
| 186650846 | rs689465 | 1 | T | PTGS2 | -0.034664 | 0.047703 | 0.46743 |
| 186774893 | rs12757858 | 1 | T | PLA2G4A | -0.003066 | 0.04345 | 0.943742 |
| 186817928 | rs1473676 | 1 | C | PLA2G4A | -0.040147 | 0.038675 | 0.299235 |
| 186870263 | rs6695515 | 1 | G | PLA2G4A | 0.015728 | 0.042156 | 0.709073 |
| 186946638 | rs2891262 | 1 | C | PLA2G4A | -0.103541 | 0.036708 | 0.004793 |
| 186957215 | rs1555204 | 1 | C | PLA2G4A | 0.051456 | 0.040468 | 0.20354 |
| 186968711 | rs12139055 | 1 | A | PLA2G4A | -0.01904 | 0.040404 | 0.637469 |
| 186982931 | rs10911985 | 1 | G | PLA2G4A | 0.014877 | 0.032175 | 0.643806 |
| 219647487 | rs4674338 | 2 | G | PRKAG3 | 0.066598 | 0.03334 | 0.045764 |
| 219713015 | rs11691497 | 2 | C | PRKAG3 | -0.096094 | 0.042182 | 0.022721 |
