# Supplementary material for: Circulating n-3 fatty acids and trans-fatty acids, PLA2G2A gene variation and sudden cardiac arrest
Source: J Nutr Sci. 2016 Mar 1;5:e12. doi: 10.1017/jns.2016.2 (PMC4791519; doi:10.1017/jns.2016.2)
Supplement: Supplementary file 1 [file S2048679016000021sup.zip › S2048679016000021sup004.docx]

Supplementary table 4: Comprehensive results for EPA

| Position | Marker | Chrom | Coded allele | gene | Beta coef | SE | p-value |
| --- | --- | --- | --- | --- | --- | --- | --- |
| 4582183 | rs55682338 | 17 | G | ALOX15 | -0.001764 | 0.009033 | 0.845193 |
| 5051532 | rs1937863 | 10 | G | AKR1C3 | -0.013625 | 0.00985 | 0.166579 |
| 5060658 | rs1937887 | 10 | T | AKR1C3 | 0.02025 | 0.01222 | 0.0975 |
| 5085931 | rs56114059 | 10 | G | AKR1C3 | -0.011996 | 0.010779 | 0.26576 |
| 5113711 | rs4881394 | 10 | T | AKR1C3 | 0.002202 | 0.008161 | 0.787285 |
| 5114183 | rs34477787 | 10 | C | AKR1C3 | -0.001601 | 0.010343 | 0.876999 |
| 5134511 | rs1937847 | 10 | A | AKR1C3 | 0.009162 | 0.0098 | 0.349878 |
| 5138607 | rs7741 | 10 | G | AKR1C3 | -0.000579 | 0.007617 | 0.939462 |
| 6899629 | rs3218667 | 17 | C | ALOX12 | -0.003924 | 0.007693 | 0.610016 |
| 6915401 | rs2271316 | 17 | C | ALOX12 | -0.002067 | 0.008171 | 0.80028 |
| 6963609 | rs11654772 | 17 | C | ALOX12 | -0.014244 | 0.008789 | 0.105087 |
| 7089652 | rs314256 | 17 | C | ACADVL | -0.001201 | 0.008256 | 0.884366 |
| 7154582 | rs3744399 | 17 | T | ACADVL | -0.003263 | 0.010605 | 0.7583 |
| 19748603 | rs7844579 | 8 | T | LPL | 0.004359 | 0.007389 | 0.555215 |
| 19797916 | rs3779787 | 8 | G | LPL | 0.003646 | 0.010451 | 0.727192 |
| 19869676 | rs1919484 | 8 | G | LPL | -0.007725 | 0.008582 | 0.368031 |
| 20273680 | rs4654990 | 1 | G | PLA2G2A | -0.019541 | 0.007899 | 0.013369 |
| 20275868 | rs12083280 | 1 | G | PLA2G2A | 0.013056 | 0.009442 | 0.166728 |
| 20301781 | rs3767221 | 1 | A | PLA2G2A | -0.003172 | 0.007848 | 0.686084 |
| 20306146 | rs11573156 | 1 | C | PLA2G2A | -0.004957 | 0.009735 | 0.610602 |
| 20334912 | rs10916689 | 1 | G | PLA2G2A | -0.008493 | 0.009464 | 0.369531 |
| 20335423 | rs4233290 | 1 | G | PLA2G2A | -0.001507 | 0.010465 | 0.885512 |
| 20357803 | rs818675 | 1 | T | PLA2G5 | -0.008227 | 0.007608 | 0.279499 |
| 20414239 | rs11573272 | 1 | A | PLA2G5 | 0.001349 | 0.007638 | 0.859799 |
| 20420414 | rs61770054 | 1 | A | PLA2G5 | -0.006565 | 0.010345 | 0.525684 |
| 20437563 | rs7518058 | 1 | T | PLA2G5 | -0.002405 | 0.008044 | 0.76493 |
| 20451812 | rs636584 | 1 | C | PLA2G5 | 0.006491 | 0.007437 | 0.382781 |
| 23790132 | rs2970884 | 4 | C | PPARGC1A | 0.001081 | 0.008331 | 0.896785 |
| 23858960 | rs11941854 | 4 | T | PPARGC1A | 0.014298 | 0.009809 | 0.144953 |
| 23859103 | rs34478957 | 4 | C | PPARGC1A | -0.0012 | 0.007664 | 0.875526 |
| 23886131 | rs35121232 | 4 | C | PPARGC1A | -0.001108 | 0.01204 | 0.926666 |
| 23886323 | rs2946385 | 4 | G | PPARGC1A | -0.006816 | 0.007236 | 0.346182 |
| 23889781 | rs2970873 | 4 | A | PPARGC1A | -0.030772 | 0.01037 | 0.003002 |
| 23934551 | rs12642645 | 4 | A | PPARGC1A | 0.008109 | 0.007443 | 0.275902 |
| 23934688 | rs6851904 | 4 | C | PPARGC1A | -0.004219 | 0.008415 | 0.616109 |
| 24380071 | rs17794681 | 14 | C | DHRS4 | -0.009 | 0.009028 | 0.318796 |
| 24610727 | rs7284722 | 22 | G | GGT5 | -0.010813 | 0.010171 | 0.287764 |
| 24946618 | rs9624495 | 22 | G | GGT1 | 0.004146 | 0.011747 | 0.724145 |
| 26465848 | rs6752314 | 2 | C | HADHB | -0.003269 | 0.010333 | 0.751745 |
| 26491392 | rs10445947 | 2 | A | HADHB | -0.002862 | 0.00811 | 0.7242 |
| 26549127 | rs11689086 | 2 | C | HADHB | -0.014081 | 0.007779 | 0.070266 |
| 27323607 | rs2322581 | 8 | G | EPHX2 | -0.006403 | 0.010391 | 0.537765 |
| 27381996 | rs721619 | 8 | C | EPHX2 | -0.017388 | 0.007666 | 0.023317 |
| 27402494 | rs4149259 | 8 | C | EPHX2 | 0.000823 | 0.010319 | 0.936432 |
| 27405576 | rs2640727 | 8 | C | EPHX2 | 0.007057 | 0.008286 | 0.394387 |
| 27450251 | rs10111053 | 8 | G | EPHX2 | 0.001056 | 0.008977 | 0.906326 |
| 31247510 | rs61947822 | 13 | T | ALOX5AP | -0.007435 | 0.012288 | 0.545121 |
| 31256268 | rs9508815 | 13 | C | ALOX5AP | 0.01271 | 0.008088 | 0.11608 |
| 31271809 | rs3000632 | 13 | A | ALOX5AP | 0.000759 | 0.007662 | 0.921038 |
| 31296538 | rs9743182 | 13 | T | ALOX5AP | -0.005704 | 0.011411 | 0.617139 |
| 31299553 | rs17222814 | 13 | G | ALOX5AP | 0.012942 | 0.012475 | 0.299519 |
| 31317878 | rs4254165 | 13 | A | ALOX5AP | 0.000138 | 0.008094 | 0.986366 |
| 31318020 | rs4360791 | 13 | G | ALOX5AP | 0.007959 | 0.007524 | 0.290167 |
| 31366810 | rs4238140 | 13 | A | ALOX5AP | -0.015207 | 0.009989 | 0.127935 |
| 31370547 | rs9315067 | 13 | G | ALOX5AP | 0.006494 | 0.008543 | 0.447196 |
| 33413176 | rs7262274 | 20 | G | GGT7 | -0.001824 | 0.007642 | 0.811309 |
| 33460366 | rs11697978 | 20 | G | GGT7 | 0.005602 | 0.009563 | 0.558043 |
| 35475913 | rs7215365 | 17 | C | ACACA | 0.007717 | 0.009903 | 0.43582 |
| 35476626 | rs17573357 | 17 | A | ACACA | -0.008368 | 0.011012 | 0.447283 |
| 35547802 | rs11653093 | 17 | C | ACACA | 0.00698 | 0.007344 | 0.341856 |
| 35754910 | rs35707017 | 17 | C | ACACA | 0.017773 | 0.008959 | 0.047274 |
| 35760940 | rs1102920 | 17 | A | ACACA | -0.01747 | 0.010106 | 0.083866 |
| 35767603 | rs829165 | 17 | T | ACACA | 0.001588 | 0.01033 | 0.877845 |
| 37395556 | rs4817761 | 21 | C | CBR1 | 0.008172 | 0.007822 | 0.296123 |
| 37443480 | rs1005696 | 21 | T | CBR1 | 0.008695 | 0.00775 | 0.261899 |
| 37443671 | rs2156406 | 21 | A | CBR1 | -0.011725 | 0.011879 | 0.323622 |
| 37443893 | rs3787728 | 21 | T | CBR1 | 0.015531 | 0.008263 | 0.060173 |
| 37494978 | rs4816519 | 21 | A | CBR1 | 0.005438 | 0.011149 | 0.625717 |
| 37495374 | rs7283730 | 21 | G | CBR1 | 0.004452 | 0.007733 | 0.564786 |
| 45869713 | rs4987106 | 10 | C | ALOX5 | 0.009644 | 0.01103 | 0.381915 |
| 45915941 | rs2099171 | 10 | C | ALOX5 | -0.003683 | 0.008525 | 0.665683 |
| 45938239 | rs2242332 | 10 | T | ALOX5 | 0.004344 | 0.008207 | 0.596577 |
| 46702601 | rs6935058 | 6 | A | PLA2G7 | -0.010564 | 0.01067 | 0.322123 |
| 46703319 | rs9395208 | 6 | G | PLA2G7 | 0.004461 | 0.009167 | 0.626537 |
| 48125015 | rs13039739 | 20 | G | PTGIS | 0.001128 | 0.009459 | 0.905103 |
| 48196962 | rs538748 | 20 | G | PTGIS | -0.001541 | 0.008184 | 0.850663 |
| 48210099 | rs508864 | 20 | C | PTGIS | -0.011728 | 0.009118 | 0.198363 |
| 48893780 | rs9840684 | 3 | T | SLC25A20 | -0.018869 | 0.008356 | 0.023939 |
| 49351654 | rs833827 | 12 | A | PRKAG1 | 0.002171 | 0.008052 | 0.787482 |
| 49389320 | rs1054442 | 12 | A | PRKAG1 | -0.00083 | 0.008162 | 0.919045 |
| 49451459 | rs833836 | 12 | C | PRKAG1 | 0.016237 | 0.010125 | 0.108795 |
| 49455006 | rs11168838 | 12 | A | PRKAG1 | -0.000141 | 0.008599 | 0.986928 |
| 53037658 | rs2452763 | 1 | G | GPX7 | 0.004745 | 0.011466 | 0.678988 |
| 53068093 | rs11205977 | 1 | G | GPX7 | 0.018373 | 0.011722 | 0.117019 |
| 53068430 | rs12089784 | 1 | G | GPX7 | 0.009233 | 0.007394 | 0.211806 |
| 53098558 | rs12728876 | 1 | A | GPX7 | -0.003496 | 0.010522 | 0.739681 |
| 53613018 | rs1679936 | 1 | G | CPT2 | 0.00563 | 0.007607 | 0.459199 |
| 53613907 | rs6696614 | 1 | A | CPT2 | -0.017112 | 0.008087 | 0.034347 |
| 53663128 | rs3766760 | 1 | G | CPT2 | -0.010098 | 0.007733 | 0.191599 |
| 53686383 | rs1679913 | 1 | C | CPT2 | -0.005974 | 0.008012 | 0.455864 |
| 57072543 | rs2796540 | 1 | G | PRKAA2 | 0.003908 | 0.007384 | 0.596628 |
| 57092231 | rs7542282 | 1 | A | PRKAA2 | -0.006315 | 0.011917 | 0.596187 |
| 57202872 | rs6656021 | 1 | A | PRKAA2 | 0.003598 | 0.009695 | 0.710532 |
| 57223201 | rs10489620 | 1 | C | PRKAA2 | -0.002526 | 0.00745 | 0.734617 |
| 57223222 | rs857105 | 1 | G | PRKAA2 | 0.001975 | 0.007968 | 0.804194 |
| 60392271 | rs11572191 | 1 | G | CYP2J2 | 0.001289 | 0.012011 | 0.914535 |
| 60413890 | rs7518613 | 1 | T | CYP2J2 | 0.017462 | 0.012134 | 0.15011 |
| 60413925 | rs11207546 | 1 | C | CYP2J2 | -0.005946 | 0.008081 | 0.461836 |
| 60436924 | rs877494 | 1 | C | CYP2J2 | 0.004182 | 0.007869 | 0.595132 |
| 71284954 | rs1327453 | 1 | G | PTGER3 | -0.002666 | 0.007735 | 0.730366 |
| 71398588 | rs12067140 | 1 | A | PTGER3 | -0.011192 | 0.011609 | 0.335004 |
| 71432984 | rs626398 | 1 | A | PTGER3 | 0.011131 | 0.010312 | 0.280404 |
| 71444355 | rs601934 | 1 | T | PTGER3 | -0.001643 | 0.007594 | 0.828673 |
| 71549987 | rs11808123 | 1 | A | PTGER3 | 0.003537 | 0.011895 | 0.766222 |
| 71556167 | rs7531139 | 1 | A | PTGER3 | -4.30E-05 | 0.009455 | 0.996351 |
| 74741181 | rs7893781 | 10 | T | PLA2G12B | 0.004332 | 0.00762 | 0.569728 |
| 76191744 | rs11161465 | 1 | T | ACADM | -0.002563 | 0.008219 | 0.75513 |
| 76252335 | rs1146635 | 1 | A | ACADM | -0.018126 | 0.007659 | 0.017951 |
| 76255228 | rs11161620 | 1 | G | ACADM | 0.022114 | 0.00846 | 0.008949 |
| 83912536 | rs12716744 | 16 | A | MLYCD | 0.004758 | 0.008173 | 0.5605 |
| 90996258 | rs1805844 | 8 | A | DECR1 | -0.002028 | 0.008023 | 0.80046 |
| 96357235 | rs7486464 | 12 | A | LTA4H | 0.013616 | 0.007744 | 0.078703 |
| 96374750 | rs2270318 | 12 | G | LTA4H | -0.006959 | 0.007566 | 0.357724 |
| 96408976 | rs2072512 | 12 | T | LTA4H | 0.004541 | 0.007452 | 0.542244 |
| 96437903 | rs2660842 | 12 | A | LTA4H | -0.011231 | 0.008622 | 0.192729 |
| 96437926 | rs2660843 | 12 | A | LTA4H | -0.01529 | 0.009341 | 0.101668 |
| 96451981 | rs4762661 | 12 | T | LTA4H | -0.003278 | 0.010588 | 0.756879 |
| 96455871 | rs2660885 | 12 | C | LTA4H | -0.003394 | 0.009443 | 0.719252 |
| 96464620 | rs7306046 | 12 | A | LTA4H | -0.007162 | 0.009358 | 0.444105 |
| 96485986 | rs58006709 | 12 | C | LTA4H | -0.003476 | 0.010593 | 0.742818 |
| 107947457 | rs7120711 | 11 | G | ACAT1 | 0.007936 | 0.00899 | 0.377374 |
| 107986691 | rs11601596 | 11 | T | ACAT1 | 0.001574 | 0.008839 | 0.8587 |
| 107992312 | rs3741054 | 11 | C | ACAT1 | -0.009015 | 0.009693 | 0.352305 |
| 108013093 | rs10890818 | 11 | A | ACAT1 | -0.013521 | 0.010484 | 0.19715 |
| 108912668 | rs6533329 | 4 | A | HADH | 0.013462 | 0.01102 | 0.221856 |
| 108931551 | rs141066 | 4 | A | HADH | 0.000566 | 0.008412 | 0.94639 |
| 109535809 | rs1018782 | 12 | A | ACACB | 0.004999 | 0.010471 | 0.633041 |
| 109549579 | rs7978946 | 12 | C | ACACB | 0.002601 | 0.009433 | 0.782756 |
| 109592483 | rs11610260 | 12 | T | ACACB | 0.004597 | 0.009215 | 0.617889 |
| 109693348 | rs55960723 | 12 | G | ACACB | 0.003642 | 0.012744 | 0.775048 |
| 109740442 | rs11066182 | 12 | G | ACACB | -0.010514 | 0.008212 | 0.200401 |
| 109740593 | rs11831762 | 12 | C | ACACB | 0.015859 | 0.011934 | 0.183892 |
| 110621820 | rs5030539 | 4 | A | PLA2G12A | 0.005056 | 0.007643 | 0.508325 |
| 110651146 | rs11555260 | 4 | C | PLA2G12A | -0.009076 | 0.007732 | 0.240491 |
| 117421595 | rs11807991 | 1 | G | PTGFRN | 0.013318 | 0.009341 | 0.153938 |
| 117487884 | rs10923173 | 1 | G | PTGFRN | -0.00094 | 0.007869 | 0.904911 |
| 117514334 | rs6687760 | 1 | A | PTGFRN | -0.018033 | 0.010535 | 0.086942 |
| 117540386 | rs2057592 | 1 | A | PTGFRN | -0.0059 | 0.00814 | 0.468535 |
| 117550327 | rs2806873 | 1 | C | PTGFRN | 0.00582 | 0.009381 | 0.534989 |
| 120097006 | rs7967558 | 12 | T | PRKAB1 | 0.008306 | 0.007883 | 0.291989 |
| 120105707 | rs6490265 | 12 | T | PRKAB1 | -0.000376 | 0.010027 | 0.970095 |
| 125139340 | rs35119072 | 9 | T | PTGS1 | -0.023976 | 0.010602 | 0.023728 |
| 125164936 | rs10513402 | 9 | T | PTGS1 | -0.013646 | 0.012576 | 0.277865 |
| 125180343 | rs2778618 | 9 | A | PTGS1 | -0.00591 | 0.010512 | 0.573973 |
| 130850583 | rs10987870 | 9 | T | PTGES2 | 0.014759 | 0.011207 | 0.187872 |
| 132523860 | rs10118377 | 9 | A | PTGES | -0.002745 | 0.008267 | 0.739885 |
| 132538919 | rs1017509 | 9 | T | PTGES | 0.004361 | 0.010777 | 0.685748 |
| 132552936 | rs11793199 | 9 | T | PTGES | -0.009236 | 0.00867 | 0.286774 |
| 139463264 | rs1011018 | 7 | G | TBXAS1 | 0.015581 | 0.009987 | 0.118722 |
| 139523858 | rs1990354 | 7 | A | TBXAS1 | 0.007624 | 0.007546 | 0.312353 |
| 139553641 | rs41706 | 7 | T | TBXAS1 | -0.00418 | 0.008352 | 0.616731 |
| 139596859 | rs2267691 | 7 | C | TBXAS1 | -0.003288 | 0.011556 | 0.77598 |
| 139609317 | rs1978180 | 7 | C | TBXAS1 | 0.014609 | 0.011054 | 0.186295 |
| 139618001 | rs2299891 | 7 | T | TBXAS1 | 0.003437 | 0.008077 | 0.670468 |
| 139640896 | rs4726473 | 7 | T | TBXAS1 | 0.004163 | 0.00845 | 0.622267 |
| 139687783 | rs2267703 | 7 | G | TBXAS1 | 0.000489 | 0.007855 | 0.950405 |
| 139695316 | rs2284212 | 7 | G | TBXAS1 | 0.007019 | 0.008252 | 0.394997 |
| 139741766 | rs10267006 | 7 | C | TBXAS1 | -0.013922 | 0.009598 | 0.146923 |
| 139761463 | rs7794528 | 7 | C | TBXAS1 | 0.005643 | 0.00878 | 0.520423 |
| 139763419 | rs6943771 | 7 | C | TBXAS1 | -0.016841 | 0.011822 | 0.15428 |
| 139822542 | rs908831 | 9 | C | PTGDS | 0.003697 | 0.007415 | 0.618036 |
| 146610792 | rs4397700 | 1 | C | PRKAB2 | 0.004413 | 0.011916 | 0.711143 |
| 146641686 | rs3766522 | 1 | A | PRKAB2 | 0.010232 | 0.008518 | 0.229663 |
| 150369314 | rs57652693 | 5 | G | GPX3 | -0.003201 | 0.010337 | 0.756846 |
| 150384959 | rs2054440 | 5 | A | GPX3 | 0.001862 | 0.007243 | 0.797112 |
| 150400587 | rs870407 | 5 | A | GPX3 | 0.000492 | 0.011031 | 0.964432 |
| 150404311 | rs8177433 | 5 | C | GPX3 | 0.003953 | 0.009756 | 0.685326 |
| 150428584 | rs4958878 | 5 | A | GPX3 | -0.017459 | 0.012164 | 0.151204 |
| 150428871 | rs6862024 | 5 | G | GPX3 | -3.00E-06 | 0.007982 | 0.999713 |
| 150455672 | rs3792784 | 5 | A | GPX3 | 0.007243 | 0.011984 | 0.545578 |
| 175411953 | rs9312555 | 4 | A | HPGD | -0.006086 | 0.010482 | 0.561528 |
| 175442822 | rs1365613 | 4 | T | HPGD | -0.009547 | 0.009944 | 0.337035 |
| 175444281 | rs17553108 | 4 | G | HPGD | 0.005358 | 0.011117 | 0.629856 |
| 175463488 | rs2256673 | 4 | T | HPGD | -0.00291 | 0.010065 | 0.772505 |
| 175463555 | rs2256669 | 4 | C | HPGD | -0.006416 | 0.009122 | 0.481837 |
| 175475048 | rs17060632 | 4 | A | HPGD | -0.001043 | 0.010722 | 0.922472 |
| 179177116 | rs55827522 | 5 | C | LTC4S | -0.014137 | 0.008213 | 0.085204 |
| 179250392 | rs10464093 | 5 | G | LTC4S | -0.002707 | 0.007885 | 0.731357 |
| 186612725 | rs10911899 | 1 | T | PTGS2 | 0.002167 | 0.011786 | 0.854144 |
| 186648197 | rs5277 | 1 | C | PTGS2 | 0.0145 | 0.009861 | 0.141433 |
| 186649221 | rs2745557 | 1 | A | PTGS2 | -0.008604 | 0.010086 | 0.393623 |
| 186650751 | rs689466 | 1 | T | PTGS2 | 0.004525 | 0.009973 | 0.650038 |
| 186650846 | rs689465 | 1 | T | PTGS2 | -0.011675 | 0.011135 | 0.294407 |
| 186774893 | rs12757858 | 1 | T | PLA2G4A | 0.003693 | 0.010143 | 0.715792 |
| 186817928 | rs1473676 | 1 | C | PLA2G4A | -0.011157 | 0.009028 | 0.216511 |
| 186870263 | rs6695515 | 1 | G | PLA2G4A | 0.008254 | 0.00984 | 0.40154 |
| 186946638 | rs2891262 | 1 | C | PLA2G4A | -0.010203 | 0.008585 | 0.234627 |
| 186957215 | rs1555204 | 1 | C | PLA2G4A | 0.014779 | 0.009445 | 0.11766 |
| 186968711 | rs12139055 | 1 | A | PLA2G4A | 0.000911 | 0.009433 | 0.923073 |
| 186982931 | rs10911985 | 1 | G | PLA2G4A | -0.00033 | 0.007512 | 0.964971 |
| 219647487 | rs4674338 | 2 | G | PRKAG3 | -0.002458 | 0.007791 | 0.752364 |
| 219713015 | rs11691497 | 2 | C | PRKAG3 | -0.005925 | 0.00986 | 0.547934 |
