# Supplementary material for: Circulating n-3 fatty acids and trans-fatty acids, PLA2G2A gene variation and sudden cardiac arrest
Source: J Nutr Sci. 2016 Mar 1;5:e12. doi: 10.1017/jns.2016.2 (PMC4791519; doi:10.1017/jns.2016.2)
Supplement: Supplementary file 1 [file S2048679016000021sup.zip › S2048679016000021sup005.docx]

Supplementary table 5: Comprehensive results for *trans*-18:1 fatty acids

| Position | Marker | Chrom | Coded allele | gene | Beta coef | SE | p-value |
| --- | --- | --- | --- | --- | --- | --- | --- |
| 4582183 | rs55682338 | 17 | G | ALOX15 | -0.026108 | 0.022362 | 0.242986 |
| 5051532 | rs1937863 | 10 | G | AKR1C3 | 0.063182 | 0.024361 | 0.009499 |
| 5060658 | rs1937887 | 10 | T | AKR1C3 | -0.04562 | 0.030266 | 0.131736 |
| 5085931 | rs56114059 | 10 | G | AKR1C3 | 0.063675 | 0.026662 | 0.016928 |
| 5113711 | rs4881394 | 10 | T | AKR1C3 | 0.01201 | 0.020209 | 0.552312 |
| 5114183 | rs34477787 | 10 | C | AKR1C3 | -0.015067 | 0.02561 | 0.556334 |
| 5134511 | rs1937847 | 10 | A | AKR1C3 | -0.015695 | 0.024273 | 0.517888 |
| 5138607 | rs7741 | 10 | G | AKR1C3 | -0.017481 | 0.01886 | 0.353962 |
| 6899629 | rs3218667 | 17 | C | ALOX12 | 0.00353 | 0.019053 | 0.853014 |
| 6915401 | rs2271316 | 17 | C | ALOX12 | 0.010067 | 0.020235 | 0.618838 |
| 6963609 | rs11654772 | 17 | C | ALOX12 | -0.031319 | 0.021769 | 0.150234 |
| 7089652 | rs314256 | 17 | C | ACADVL | 0.009884 | 0.020444 | 0.628765 |
| 7154582 | rs3744399 | 17 | T | ACADVL | 0.041482 | 0.026245 | 0.113972 |
| 19748603 | rs7844579 | 8 | T | LPL | 0.024087 | 0.018292 | 0.187893 |
| 19797916 | rs3779787 | 8 | G | LPL | 0.01255 | 0.025881 | 0.627748 |
| 19869676 | rs1919484 | 8 | G | LPL | 0.018702 | 0.021253 | 0.378858 |
| 20273680 | rs4654990 | 1 | G | PLA2G2A | 0.054576 | 0.019553 | 0.005252 |
| 20275868 | rs12083280 | 1 | G | PLA2G2A | -0.023747 | 0.023388 | 0.309926 |
| 20301781 | rs3767221 | 1 | A | PLA2G2A | 0.02688 | 0.019426 | 0.16645 |
| 20306146 | rs11573156 | 1 | C | PLA2G2A | 0.023738 | 0.024103 | 0.324685 |
| 20334912 | rs10916689 | 1 | G | PLA2G2A | 0.03579 | 0.023427 | 0.126587 |
| 20335423 | rs4233290 | 1 | G | PLA2G2A | -0.025327 | 0.025908 | 0.328283 |
| 20357803 | rs818675 | 1 | T | PLA2G5 | 0.021617 | 0.018839 | 0.251202 |
| 20414239 | rs11573272 | 1 | A | PLA2G5 | -0.019728 | 0.018909 | 0.296819 |
| 20420414 | rs61770054 | 1 | A | PLA2G5 | 0.010344 | 0.02562 | 0.686412 |
| 20437563 | rs7518058 | 1 | T | PLA2G5 | 0.019485 | 0.019915 | 0.327888 |
| 20451812 | rs636584 | 1 | C | PLA2G5 | -0.002561 | 0.01842 | 0.889421 |
| 23790132 | rs2970884 | 4 | C | PPARGC1A | -0.006152 | 0.020631 | 0.765569 |
| 23858960 | rs11941854 | 4 | T | PPARGC1A | -0.038236 | 0.02429 | 0.115451 |
| 23859103 | rs34478957 | 4 | C | PPARGC1A | -0.008658 | 0.018977 | 0.648237 |
| 23886131 | rs35121232 | 4 | C | PPARGC1A | -0.014601 | 0.029815 | 0.624344 |
| 23886323 | rs2946385 | 4 | G | PPARGC1A | 0.01561 | 0.017919 | 0.383678 |
| 23889781 | rs2970873 | 4 | A | PPARGC1A | 0.00188 | 0.02574 | 0.941786 |
| 23934551 | rs12642645 | 4 | A | PPARGC1A | -0.004012 | 0.018437 | 0.827742 |
| 23934688 | rs6851904 | 4 | C | PPARGC1A | 0.026632 | 0.02083 | 0.201067 |
| 24380071 | rs17794681 | 14 | C | DHRS4 | 0.009415 | 0.022361 | 0.673724 |
| 24610727 | rs7284722 | 22 | G | GGT5 | 0.014278 | 0.025194 | 0.570908 |
| 24946618 | rs9624495 | 22 | G | GGT1 | 0.047867 | 0.02907 | 0.099639 |
| 26465848 | rs6752314 | 2 | C | HADHB | 0.013437 | 0.025588 | 0.599493 |
| 26491392 | rs10445947 | 2 | A | HADHB | 0.025512 | 0.020076 | 0.203823 |
| 26549127 | rs11689086 | 2 | C | HADHB | 0.010246 | 0.019279 | 0.595086 |
| 27323607 | rs2322581 | 8 | G | EPHX2 | -0.014212 | 0.025732 | 0.580744 |
| 27381996 | rs721619 | 8 | C | EPHX2 | 0.004178 | 0.01901 | 0.826058 |
| 27402494 | rs4149259 | 8 | C | EPHX2 | 0.030544 | 0.025544 | 0.231788 |
| 27405576 | rs2640727 | 8 | C | EPHX2 | 0.013611 | 0.020521 | 0.507155 |
| 27450251 | rs10111053 | 8 | G | EPHX2 | 0.00618 | 0.02223 | 0.780999 |
| 31247510 | rs61947822 | 13 | T | ALOX5AP | -0.002206 | 0.030433 | 0.942226 |
| 31256268 | rs9508815 | 13 | C | ALOX5AP | 0.006022 | 0.020042 | 0.763802 |
| 31271809 | rs3000632 | 13 | A | ALOX5AP | -0.018204 | 0.018969 | 0.337233 |
| 31296538 | rs9743182 | 13 | T | ALOX5AP | 0.012383 | 0.028258 | 0.66122 |
| 31299553 | rs17222814 | 13 | G | ALOX5AP | -0.011547 | 0.030901 | 0.708652 |
| 31317878 | rs4254165 | 13 | A | ALOX5AP | 0.005289 | 0.020043 | 0.791871 |
| 31318020 | rs4360791 | 13 | G | ALOX5AP | -0.006322 | 0.018639 | 0.734459 |
| 31366810 | rs4238140 | 13 | A | ALOX5AP | 0.043132 | 0.024733 | 0.081171 |
| 31370547 | rs9315067 | 13 | G | ALOX5AP | 0.025345 | 0.021152 | 0.230823 |
| 33413176 | rs7262274 | 20 | G | GGT7 | -0.007909 | 0.018924 | 0.675995 |
| 33460366 | rs11697978 | 20 | G | GGT7 | 0.000669 | 0.023684 | 0.977461 |
| 35475913 | rs7215365 | 17 | C | ACACA | 0.044583 | 0.024506 | 0.068868 |
| 35476626 | rs17573357 | 17 | A | ACACA | 0.013151 | 0.027272 | 0.629651 |
| 35547802 | rs11653093 | 17 | C | ACACA | 0.022072 | 0.018184 | 0.22481 |
| 35754910 | rs35707017 | 17 | C | ACACA | 0.009331 | 0.022208 | 0.674379 |
| 35760940 | rs1102920 | 17 | A | ACACA | 0.022397 | 0.025042 | 0.371107 |
| 35767603 | rs829165 | 17 | T | ACACA | -0.012842 | 0.025579 | 0.61564 |
| 37395556 | rs4817761 | 21 | C | CBR1 | -0.035929 | 0.019358 | 0.063455 |
| 37443480 | rs1005696 | 21 | T | CBR1 | 0.001414 | 0.019198 | 0.9413 |
| 37443671 | rs2156406 | 21 | A | CBR1 | 0.027667 | 0.029418 | 0.346961 |
| 37443893 | rs3787728 | 21 | T | CBR1 | -0.01134 | 0.020481 | 0.579807 |
| 37494978 | rs4816519 | 21 | A | CBR1 | 0.010476 | 0.02761 | 0.704362 |
| 37495374 | rs7283730 | 21 | G | CBR1 | 0.004947 | 0.01915 | 0.79615 |
| 45869713 | rs4987106 | 10 | C | ALOX5 | -0.008498 | 0.02732 | 0.755765 |
| 45915941 | rs2099171 | 10 | C | ALOX5 | -0.006857 | 0.021112 | 0.745326 |
| 45938239 | rs2242332 | 10 | T | ALOX5 | -0.002527 | 0.020325 | 0.901066 |
| 46702601 | rs6935058 | 6 | A | PLA2G7 | 0.045626 | 0.026409 | 0.08405 |
| 46703319 | rs9395208 | 6 | G | PLA2G7 | -0.000146 | 0.022703 | 0.99487 |
| 48125015 | rs13039739 | 20 | G | PTGIS | 0.024616 | 0.023417 | 0.293166 |
| 48196962 | rs538748 | 20 | G | PTGIS | -0.012844 | 0.020266 | 0.526232 |
| 48210099 | rs508864 | 20 | C | PTGIS | 0.02792 | 0.022581 | 0.216295 |
| 48893780 | rs9840684 | 3 | T | SLC25A20 | 0.015416 | 0.020718 | 0.456836 |
| 49351654 | rs833827 | 12 | A | PRKAG1 | 0.011738 | 0.019939 | 0.556053 |
| 49389320 | rs1054442 | 12 | A | PRKAG1 | -0.019231 | 0.020206 | 0.341246 |
| 49451459 | rs833836 | 12 | C | PRKAG1 | -0.00427 | 0.025091 | 0.864857 |
| 49455006 | rs11168838 | 12 | A | PRKAG1 | -0.02893 | 0.021284 | 0.174085 |
| 53037658 | rs2452763 | 1 | G | GPX7 | 0.007563 | 0.028396 | 0.789961 |
| 53068093 | rs11205977 | 1 | G | GPX7 | -0.054156 | 0.02902 | 0.062019 |
| 53068430 | rs12089784 | 1 | G | GPX7 | -0.019442 | 0.018313 | 0.288392 |
| 53098558 | rs12728876 | 1 | A | GPX7 | 0.013258 | 0.026057 | 0.610881 |
| 53613018 | rs1679936 | 1 | G | CPT2 | -0.020633 | 0.018834 | 0.27327 |
| 53613907 | rs6696614 | 1 | A | CPT2 | 0.038364 | 0.020031 | 0.055465 |
| 53663128 | rs3766760 | 1 | G | CPT2 | -0.002266 | 0.019159 | 0.90586 |
| 53686383 | rs1679913 | 1 | C | CPT2 | -0.001353 | 0.019844 | 0.945651 |
| 57072543 | rs2796540 | 1 | G | PRKAA2 | -0.031565 | 0.018273 | 0.084091 |
| 57092231 | rs7542282 | 1 | A | PRKAA2 | -0.005943 | 0.029513 | 0.840401 |
| 57202872 | rs6656021 | 1 | A | PRKAA2 | 0.013428 | 0.024007 | 0.575943 |
| 57223201 | rs10489620 | 1 | C | PRKAA2 | -0.025122 | 0.018441 | 0.173109 |
| 57223222 | rs857105 | 1 | G | PRKAA2 | 0.008842 | 0.019731 | 0.654075 |
| 60392271 | rs11572191 | 1 | G | CYP2J2 | -0.002598 | 0.029744 | 0.930387 |
| 60413890 | rs7518613 | 1 | T | CYP2J2 | 0.005441 | 0.030064 | 0.856383 |
| 60413925 | rs11207546 | 1 | C | CYP2J2 | 0.022241 | 0.020008 | 0.266298 |
| 60436924 | rs877494 | 1 | C | CYP2J2 | -0.002419 | 0.019489 | 0.901206 |
| 71284954 | rs1327453 | 1 | G | PTGER3 | 0.02284 | 0.019147 | 0.232929 |
| 71398588 | rs12067140 | 1 | A | PTGER3 | -0.036069 | 0.028743 | 0.209525 |
| 71432984 | rs626398 | 1 | A | PTGER3 | 0.01257 | 0.025544 | 0.622657 |
| 71444355 | rs601934 | 1 | T | PTGER3 | -0.007287 | 0.018806 | 0.698404 |
| 71549987 | rs11808123 | 1 | A | PTGER3 | 0.025781 | 0.029451 | 0.381377 |
| 71556167 | rs7531139 | 1 | A | PTGER3 | -0.023025 | 0.023408 | 0.325291 |
| 74741181 | rs7893781 | 10 | T | PLA2G12B | 0.000124 | 0.018873 | 0.994775 |
| 76191744 | rs11161465 | 1 | T | ACADM | -0.019524 | 0.020348 | 0.337309 |
| 76252335 | rs1146635 | 1 | A | ACADM | 0.009581 | 0.018995 | 0.613991 |
| 76255228 | rs11161620 | 1 | G | ACADM | -0.035075 | 0.020973 | 0.094444 |
| 83912536 | rs12716744 | 16 | A | MLYCD | 0.013289 | 0.02024 | 0.511446 |
| 90996258 | rs1805844 | 8 | A | DECR1 | 0.001791 | 0.019867 | 0.928183 |
| 96357235 | rs7486464 | 12 | A | LTA4H | 0.006309 | 0.019192 | 0.742358 |
| 96374750 | rs2270318 | 12 | G | LTA4H | 0.000955 | 0.018741 | 0.959352 |
| 96408976 | rs2072512 | 12 | T | LTA4H | 0.020145 | 0.018449 | 0.274883 |
| 96437903 | rs2660842 | 12 | A | LTA4H | 0.021623 | 0.021356 | 0.311302 |
| 96437926 | rs2660843 | 12 | A | LTA4H | 0.040357 | 0.02313 | 0.081024 |
| 96451981 | rs4762661 | 12 | T | LTA4H | 0.039409 | 0.026205 | 0.132614 |
| 96455871 | rs2660885 | 12 | C | LTA4H | 0.002108 | 0.023386 | 0.928176 |
| 96464620 | rs7306046 | 12 | A | LTA4H | 0.012603 | 0.023176 | 0.586585 |
| 96485986 | rs58006709 | 12 | C | LTA4H | 0.001779 | 0.026234 | 0.945936 |
| 107947457 | rs7120711 | 11 | G | ACAT1 | -0.034213 | 0.022253 | 0.124179 |
| 107986691 | rs11601596 | 11 | T | ACAT1 | 0.004702 | 0.021889 | 0.829899 |
| 107992312 | rs3741054 | 11 | C | ACAT1 | -0.033194 | 0.023996 | 0.16657 |
| 108013093 | rs10890818 | 11 | A | ACAT1 | 0.042129 | 0.025956 | 0.104568 |
| 108912668 | rs6533329 | 4 | A | HADH | 0.017782 | 0.027298 | 0.514789 |
| 108931551 | rs141066 | 4 | A | HADH | 0.004229 | 0.02083 | 0.839128 |
| 109535809 | rs1018782 | 12 | A | ACACB | -0.007014 | 0.025932 | 0.786791 |
| 109549579 | rs7978946 | 12 | C | ACACB | -0.024718 | 0.023353 | 0.289855 |
| 109592483 | rs11610260 | 12 | T | ACACB | -0.023845 | 0.022815 | 0.295951 |
| 109693348 | rs55960723 | 12 | G | ACACB | 0.00866 | 0.031558 | 0.783771 |
| 109740442 | rs11066182 | 12 | G | ACACB | 0.012613 | 0.020343 | 0.535226 |
| 109740593 | rs11831762 | 12 | C | ACACB | -0.019817 | 0.029565 | 0.502669 |
| 110621820 | rs5030539 | 4 | A | PLA2G12A | -0.012375 | 0.018928 | 0.513229 |
| 110651146 | rs11555260 | 4 | C | PLA2G12A | 0.023169 | 0.019148 | 0.226292 |
| 117421595 | rs11807991 | 1 | G | PTGFRN | -0.018303 | 0.02314 | 0.428967 |
| 117487884 | rs10923173 | 1 | G | PTGFRN | 0.006443 | 0.019485 | 0.740914 |
| 117514334 | rs6687760 | 1 | A | PTGFRN | 0.020713 | 0.026105 | 0.42752 |
| 117540386 | rs2057592 | 1 | A | PTGFRN | 0.000819 | 0.020161 | 0.967585 |
| 117550327 | rs2806873 | 1 | C | PTGFRN | 0.003678 | 0.023233 | 0.8742 |
| 120097006 | rs7967558 | 12 | T | PRKAB1 | -0.006288 | 0.019526 | 0.747433 |
| 120105707 | rs6490265 | 12 | T | PRKAB1 | 0.032639 | 0.024819 | 0.188486 |
| 125139340 | rs35119072 | 9 | T | PTGS1 | 0.059027 | 0.026254 | 0.024559 |
| 125164936 | rs10513402 | 9 | T | PTGS1 | 0.072042 | 0.031108 | 0.020566 |
| 125180343 | rs2778618 | 9 | A | PTGS1 | 0.001153 | 0.026035 | 0.964668 |
| 130850583 | rs10987870 | 9 | T | PTGES2 | -0.019369 | 0.027763 | 0.485395 |
| 132523860 | rs10118377 | 9 | A | PTGES | 0.003652 | 0.020473 | 0.858439 |
| 132538919 | rs1017509 | 9 | T | PTGES | 0.015448 | 0.026687 | 0.562674 |
| 132552936 | rs11793199 | 9 | T | PTGES | 0.036276 | 0.021462 | 0.090979 |
| 139463264 | rs1011018 | 7 | G | TBXAS1 | 0.03315 | 0.024736 | 0.180201 |
| 139523858 | rs1990354 | 7 | A | TBXAS1 | -0.029281 | 0.018681 | 0.117001 |
| 139553641 | rs41706 | 7 | T | TBXAS1 | 0.017751 | 0.02068 | 0.390693 |
| 139596859 | rs2267691 | 7 | C | TBXAS1 | 0.008383 | 0.028616 | 0.769552 |
| 139609317 | rs1978180 | 7 | C | TBXAS1 | -0.01684 | 0.027384 | 0.538599 |
| 139618001 | rs2299891 | 7 | T | TBXAS1 | -0.004086 | 0.020003 | 0.838138 |
| 139640896 | rs4726473 | 7 | T | TBXAS1 | 0.004457 | 0.020927 | 0.831327 |
| 139687783 | rs2267703 | 7 | G | TBXAS1 | 0.023382 | 0.019445 | 0.229185 |
| 139695316 | rs2284212 | 7 | G | TBXAS1 | -0.001082 | 0.02044 | 0.9578 |
| 139741766 | rs10267006 | 7 | C | TBXAS1 | 0.015902 | 0.02378 | 0.50368 |
| 139761463 | rs7794528 | 7 | C | TBXAS1 | -0.002149 | 0.021746 | 0.921292 |
| 139763419 | rs6943771 | 7 | C | TBXAS1 | 0.007286 | 0.029291 | 0.803557 |
| 139822542 | rs908831 | 9 | C | PTGDS | -0.011452 | 0.018361 | 0.532834 |
| 146610792 | rs4397700 | 1 | C | PRKAB2 | 0.046387 | 0.02949 | 0.115723 |
| 146641686 | rs3766522 | 1 | A | PRKAB2 | -0.034759 | 0.021087 | 0.099277 |
| 150369314 | rs57652693 | 5 | G | GPX3 | -0.024218 | 0.025593 | 0.344012 |
| 150384959 | rs2054440 | 5 | A | GPX3 | 0.007076 | 0.017936 | 0.693198 |
| 150400587 | rs870407 | 5 | A | GPX3 | -0.035465 | 0.027304 | 0.193982 |
| 150404311 | rs8177433 | 5 | C | GPX3 | -0.045275 | 0.024139 | 0.060711 |
| 150428584 | rs4958878 | 5 | A | GPX3 | 0.056201 | 0.030112 | 0.061989 |
| 150428871 | rs6862024 | 5 | G | GPX3 | 0.014604 | 0.019764 | 0.459963 |
| 150455672 | rs3792784 | 5 | A | GPX3 | -0.059727 | 0.029648 | 0.043957 |
| 175411953 | rs9312555 | 4 | A | HPGD | 0.021108 | 0.025955 | 0.416084 |
| 175442822 | rs1365613 | 4 | T | HPGD | 0.022824 | 0.024627 | 0.354032 |
| 175444281 | rs17553108 | 4 | G | HPGD | -0.046491 | 0.027512 | 0.091058 |
| 175463488 | rs2256673 | 4 | T | HPGD | 0.000495 | 0.024924 | 0.984141 |
| 175463555 | rs2256669 | 4 | C | HPGD | 0.013489 | 0.022591 | 0.550462 |
| 175475048 | rs17060632 | 4 | A | HPGD | -0.018254 | 0.026548 | 0.491711 |
| 179177116 | rs55827522 | 5 | C | LTC4S | 0.001342 | 0.020356 | 0.94742 |
| 179250392 | rs10464093 | 5 | G | LTC4S | -0.009958 | 0.019526 | 0.610045 |
| 186612725 | rs10911899 | 1 | T | PTGS2 | -0.033377 | 0.029176 | 0.252639 |
| 186648197 | rs5277 | 1 | C | PTGS2 | 0.025608 | 0.024427 | 0.294476 |
| 186649221 | rs2745557 | 1 | A | PTGS2 | -0.014241 | 0.024979 | 0.568599 |
| 186650751 | rs689466 | 1 | T | PTGS2 | -0.019284 | 0.024693 | 0.434851 |
| 186650846 | rs689465 | 1 | T | PTGS2 | 0.034934 | 0.02757 | 0.205125 |
| 186774893 | rs12757858 | 1 | T | PLA2G4A | -0.039576 | 0.025103 | 0.114896 |
| 186817928 | rs1473676 | 1 | C | PLA2G4A | 0.028536 | 0.022356 | 0.201798 |
| 186870263 | rs6695515 | 1 | G | PLA2G4A | 0.029781 | 0.024363 | 0.22156 |
| 186946638 | rs2891262 | 1 | C | PLA2G4A | 0.013417 | 0.021265 | 0.528092 |
| 186957215 | rs1555204 | 1 | C | PLA2G4A | 0.014763 | 0.023403 | 0.528157 |
| 186968711 | rs12139055 | 1 | A | PLA2G4A | -0.00372 | 0.02336 | 0.873481 |
| 186982931 | rs10911985 | 1 | G | PLA2G4A | 0.012085 | 0.0186 | 0.515861 |
| 219647487 | rs4674338 | 2 | G | PRKAG3 | -0.008072 | 0.019294 | 0.675671 |
| 219713015 | rs11691497 | 2 | C | PRKAG3 | 0.024153 | 0.024414 | 0.32252 |
