# Supplementary material for: Circulating n-3 fatty acids and trans-fatty acids, PLA2G2A gene variation and sudden cardiac arrest
Source: J Nutr Sci. 2016 Mar 1;5:e12. doi: 10.1017/jns.2016.2 (PMC4791519; doi:10.1017/jns.2016.2)
Supplement: Supplementary file 1 [file S2048679016000021sup.zip › S2048679016000021sup006.docx]

Supplementary table 6: Comprehensive results for *trans*-18:2 fatty acids

| Position | Marker | Chrom | Coded allele | gene | Beta coef | SE | p-value |
| --- | --- | --- | --- | --- | --- | --- | --- |
| 4582183 | rs55682338 | 17 | G | ALOX15 | -0.004215 | 0.002269 | 0.063201 |
| 5051532 | rs1937863 | 10 | G | AKR1C3 | 0.002155 | 0.002477 | 0.384347 |
| 5060658 | rs1937887 | 10 | T | AKR1C3 | 0.000653 | 0.003074 | 0.831681 |
| 5085931 | rs56114059 | 10 | G | AKR1C3 | 0.000946 | 0.002711 | 0.727195 |
| 5113711 | rs4881394 | 10 | T | AKR1C3 | 0.002822 | 0.002051 | 0.168841 |
| 5114183 | rs34477787 | 10 | C | AKR1C3 | -0.002214 | 0.0026 | 0.394416 |
| 5134511 | rs1937847 | 10 | A | AKR1C3 | 0.001703 | 0.002464 | 0.489494 |
| 5138607 | rs7741 | 10 | G | AKR1C3 | -0.005042 | 0.001911 | 0.008344 |
| 6899629 | rs3218667 | 17 | C | ALOX12 | 0.001473 | 0.001934 | 0.446297 |
| 6915401 | rs2271316 | 17 | C | ALOX12 | 0.003131 | 0.002053 | 0.12724 |
| 6963609 | rs11654772 | 17 | C | ALOX12 | -0.001487 | 0.002211 | 0.501343 |
| 7089652 | rs314256 | 17 | C | ACADVL | -0.001053 | 0.002075 | 0.611913 |
| 7154582 | rs3744399 | 17 | T | ACADVL | 0.001295 | 0.002666 | 0.627002 |
| 19748603 | rs7844579 | 8 | T | LPL | -0.000593 | 0.001858 | 0.749494 |
| 19797916 | rs3779787 | 8 | G | LPL | -0.0032 | 0.002626 | 0.223043 |
| 19869676 | rs1919484 | 8 | G | LPL | 0.000646 | 0.002158 | 0.764668 |
| 20273680 | rs4654990 | 1 | G | PLA2G2A | 0.003983 | 0.001987 | 0.045034 |
| 20275868 | rs12083280 | 1 | G | PLA2G2A | -0.001898 | 0.002374 | 0.423974 |
| 20301781 | rs3767221 | 1 | A | PLA2G2A | 0.000145 | 0.001973 | 0.941295 |
| 20306146 | rs11573156 | 1 | C | PLA2G2A | 0.002395 | 0.002447 | 0.327599 |
| 20334912 | rs10916689 | 1 | G | PLA2G2A | 0.001963 | 0.002379 | 0.409322 |
| 20335423 | rs4233290 | 1 | G | PLA2G2A | -0.003673 | 0.002629 | 0.16243 |
| 20357803 | rs818675 | 1 | T | PLA2G5 | 0.000629 | 0.001913 | 0.74244 |
| 20414239 | rs11573272 | 1 | A | PLA2G5 | -0.00115 | 0.00192 | 0.549142 |
| 20420414 | rs61770054 | 1 | A | PLA2G5 | 0.001963 | 0.002601 | 0.450373 |
| 20437563 | rs7518058 | 1 | T | PLA2G5 | -0.000735 | 0.002022 | 0.716346 |
| 20451812 | rs636584 | 1 | C | PLA2G5 | -0.001702 | 0.00187 | 0.362649 |
| 23790132 | rs2970884 | 4 | C | PPARGC1A | -0.000489 | 0.002094 | 0.815496 |
| 23858960 | rs11941854 | 4 | T | PPARGC1A | -0.00324 | 0.002466 | 0.188984 |
| 23859103 | rs34478957 | 4 | C | PPARGC1A | -0.000124 | 0.001927 | 0.948761 |
| 23886131 | rs35121232 | 4 | C | PPARGC1A | -0.003306 | 0.003026 | 0.274607 |
| 23886323 | rs2946385 | 4 | G | PPARGC1A | -0.000931 | 0.001819 | 0.608694 |
| 23889781 | rs2970873 | 4 | A | PPARGC1A | 0.001793 | 0.002613 | 0.492614 |
| 23934551 | rs12642645 | 4 | A | PPARGC1A | -0.003602 | 0.00187 | 0.054045 |
| 23934688 | rs6851904 | 4 | C | PPARGC1A | 0.004148 | 0.002113 | 0.049678 |
| 24380071 | rs17794681 | 14 | C | DHRS4 | -8.00E-05 | 0.00227 | 0.971953 |
| 24610727 | rs7284722 | 22 | G | GGT5 | 0.001954 | 0.002557 | 0.44494 |
| 24946618 | rs9624495 | 22 | G | GGT1 | -0.002133 | 0.002953 | 0.469995 |
| 26465848 | rs6752314 | 2 | C | HADHB | -0.003054 | 0.002597 | 0.239514 |
| 26491392 | rs10445947 | 2 | A | HADHB | -0.000707 | 0.002039 | 0.728943 |
| 26549127 | rs11689086 | 2 | C | HADHB | -0.001199 | 0.001957 | 0.540138 |
| 27323607 | rs2322581 | 8 | G | EPHX2 | -0.001486 | 0.002612 | 0.569406 |
| 27381996 | rs721619 | 8 | C | EPHX2 | 0.000214 | 0.00193 | 0.911755 |
| 27402494 | rs4149259 | 8 | C | EPHX2 | 0.00414 | 0.002592 | 0.110298 |
| 27405576 | rs2640727 | 8 | C | EPHX2 | 0.002569 | 0.002083 | 0.217458 |
| 27450251 | rs10111053 | 8 | G | EPHX2 | 0.001036 | 0.002257 | 0.646149 |
| 31247510 | rs61947822 | 13 | T | ALOX5AP | -0.002446 | 0.003089 | 0.4285 |
| 31256268 | rs9508815 | 13 | C | ALOX5AP | 0.002506 | 0.002034 | 0.217931 |
| 31271809 | rs3000632 | 13 | A | ALOX5AP | 0.001809 | 0.001926 | 0.347505 |
| 31296538 | rs9743182 | 13 | T | ALOX5AP | 0.003357 | 0.002868 | 0.241796 |
| 31299553 | rs17222814 | 13 | G | ALOX5AP | 0.002732 | 0.003136 | 0.383766 |
| 31317878 | rs4254165 | 13 | A | ALOX5AP | 0.004663 | 0.002032 | 0.021732 |
| 31318020 | rs4360791 | 13 | G | ALOX5AP | 0.001947 | 0.001892 | 0.303356 |
| 31366810 | rs4238140 | 13 | A | ALOX5AP | 0.002563 | 0.002512 | 0.307533 |
| 31370547 | rs9315067 | 13 | G | ALOX5AP | 0.001572 | 0.002148 | 0.464181 |
| 33413176 | rs7262274 | 20 | G | GGT7 | 0.002398 | 0.00192 | 0.211682 |
| 33460366 | rs11697978 | 20 | G | GGT7 | -0.000987 | 0.002404 | 0.68149 |
| 35475913 | rs7215365 | 17 | C | ACACA | 0.00277 | 0.002489 | 0.26585 |
| 35476626 | rs17573357 | 17 | A | ACACA | 0.002719 | 0.002768 | 0.325973 |
| 35547802 | rs11653093 | 17 | C | ACACA | -0.001361 | 0.001846 | 0.461015 |
| 35754910 | rs35707017 | 17 | C | ACACA | 0.000568 | 0.002255 | 0.801068 |
| 35760940 | rs1102920 | 17 | A | ACACA | -0.001284 | 0.002543 | 0.613667 |
| 35767603 | rs829165 | 17 | T | ACACA | -0.001191 | 0.002597 | 0.646559 |
| 37395556 | rs4817761 | 21 | C | CBR1 | -0.001362 | 0.001967 | 0.488735 |
| 37443480 | rs1005696 | 21 | T | CBR1 | 0.000481 | 0.001949 | 0.80518 |
| 37443671 | rs2156406 | 21 | A | CBR1 | 0.003358 | 0.002986 | 0.26072 |
| 37443893 | rs3787728 | 21 | T | CBR1 | -0.001075 | 0.002079 | 0.605093 |
| 37494978 | rs4816519 | 21 | A | CBR1 | 0.00247 | 0.002802 | 0.378027 |
| 37495374 | rs7283730 | 21 | G | CBR1 | -9.50E-05 | 0.001944 | 0.961061 |
| 45869713 | rs4987106 | 10 | C | ALOX5 | 0.000751 | 0.002773 | 0.78665 |
| 45915941 | rs2099171 | 10 | C | ALOX5 | -0.001157 | 0.002143 | 0.589216 |
| 45938239 | rs2242332 | 10 | T | ALOX5 | -0.000333 | 0.002063 | 0.871842 |
| 46702601 | rs6935058 | 6 | A | PLA2G7 | -0.000606 | 0.002683 | 0.821209 |
| 46703319 | rs9395208 | 6 | G | PLA2G7 | 0.002451 | 0.002304 | 0.287392 |
| 48125015 | rs13039739 | 20 | G | PTGIS | 0.002437 | 0.002377 | 0.30537 |
| 48196962 | rs538748 | 20 | G | PTGIS | 0.000393 | 0.002057 | 0.848502 |
| 48210099 | rs508864 | 20 | C | PTGIS | -0.000638 | 0.002293 | 0.780812 |
| 48893780 | rs9840684 | 3 | T | SLC25A20 | 0.002847 | 0.002103 | 0.175704 |
| 49351654 | rs833827 | 12 | A | PRKAG1 | -0.000941 | 0.002024 | 0.642085 |
| 49389320 | rs1054442 | 12 | A | PRKAG1 | -0.000517 | 0.002052 | 0.801038 |
| 49451459 | rs833836 | 12 | C | PRKAG1 | 0.001871 | 0.002547 | 0.462565 |
| 49455006 | rs11168838 | 12 | A | PRKAG1 | -0.002091 | 0.002161 | 0.333249 |
| 53037658 | rs2452763 | 1 | G | GPX7 | 0.001547 | 0.002882 | 0.59136 |
| 53068093 | rs11205977 | 1 | G | GPX7 | -0.006076 | 0.002945 | 0.039109 |
| 53068430 | rs12089784 | 1 | G | GPX7 | -0.00195 | 0.001859 | 0.294255 |
| 53098558 | rs12728876 | 1 | A | GPX7 | 0.001803 | 0.002645 | 0.49552 |
| 53613018 | rs1679936 | 1 | G | CPT2 | -0.000227 | 0.001913 | 0.905532 |
| 53613907 | rs6696614 | 1 | A | CPT2 | -0.000119 | 0.002035 | 0.95339 |
| 53663128 | rs3766760 | 1 | G | CPT2 | 0.003789 | 0.001943 | 0.051148 |
| 53686383 | rs1679913 | 1 | C | CPT2 | -0.003228 | 0.002013 | 0.108858 |
| 57072543 | rs2796540 | 1 | G | PRKAA2 | -0.00044 | 0.001856 | 0.812443 |
| 57092231 | rs7542282 | 1 | A | PRKAA2 | 0.002528 | 0.002996 | 0.398727 |
| 57202872 | rs6656021 | 1 | A | PRKAA2 | -0.000222 | 0.002437 | 0.92731 |
| 57223201 | rs10489620 | 1 | C | PRKAA2 | -0.002224 | 0.001872 | 0.234959 |
| 57223222 | rs857105 | 1 | G | PRKAA2 | 0.001504 | 0.002003 | 0.452723 |
| 60392271 | rs11572191 | 1 | G | CYP2J2 | 0.000849 | 0.003019 | 0.778457 |
| 60413890 | rs7518613 | 1 | T | CYP2J2 | -0.002334 | 0.003052 | 0.444393 |
| 60413925 | rs11207546 | 1 | C | CYP2J2 | 0.00244 | 0.002031 | 0.229583 |
| 60436924 | rs877494 | 1 | C | CYP2J2 | 0.000921 | 0.001978 | 0.641477 |
| 71284954 | rs1327453 | 1 | G | PTGER3 | 0.000588 | 0.001944 | 0.762319 |
| 71398588 | rs12067140 | 1 | A | PTGER3 | -0.003906 | 0.002918 | 0.180633 |
| 71432984 | rs626398 | 1 | A | PTGER3 | -0.00074 | 0.002593 | 0.775489 |
| 71444355 | rs601934 | 1 | T | PTGER3 | -0.001227 | 0.001909 | 0.520281 |
| 71549987 | rs11808123 | 1 | A | PTGER3 | 0.00108 | 0.00299 | 0.717991 |
| 71556167 | rs7531139 | 1 | A | PTGER3 | -0.001845 | 0.002376 | 0.437551 |
| 74741181 | rs7893781 | 10 | T | PLA2G12B | 6.70E-05 | 0.001916 | 0.972045 |
| 76191744 | rs11161465 | 1 | T | ACADM | -2.80E-05 | 0.002066 | 0.989076 |
| 76252335 | rs1146635 | 1 | A | ACADM | 0.00263 | 0.001927 | 0.172404 |
| 76255228 | rs11161620 | 1 | G | ACADM | -0.00056 | 0.002131 | 0.792546 |
| 83912536 | rs12716744 | 16 | A | MLYCD | 0.001956 | 0.002054 | 0.34102 |
| 90996258 | rs1805844 | 8 | A | DECR1 | 0.00011 | 0.002017 | 0.956458 |
| 96357235 | rs7486464 | 12 | A | LTA4H | 0.001232 | 0.001948 | 0.527127 |
| 96374750 | rs2270318 | 12 | G | LTA4H | -0.001526 | 0.001902 | 0.422492 |
| 96408976 | rs2072512 | 12 | T | LTA4H | 0.001309 | 0.001873 | 0.484755 |
| 96437903 | rs2660842 | 12 | A | LTA4H | 0.000916 | 0.002168 | 0.672681 |
| 96437926 | rs2660843 | 12 | A | LTA4H | 0.003654 | 0.002348 | 0.119767 |
| 96451981 | rs4762661 | 12 | T | LTA4H | 0.002133 | 0.002661 | 0.422919 |
| 96455871 | rs2660885 | 12 | C | LTA4H | -0.000136 | 0.002374 | 0.954307 |
| 96464620 | rs7306046 | 12 | A | LTA4H | 0.000413 | 0.002353 | 0.860606 |
| 96485986 | rs58006709 | 12 | C | LTA4H | 0.001463 | 0.002663 | 0.582656 |
| 107947457 | rs7120711 | 11 | G | ACAT1 | 0.000191 | 0.00226 | 0.932678 |
| 107986691 | rs11601596 | 11 | T | ACAT1 | 0.000368 | 0.002222 | 0.868567 |
| 107992312 | rs3741054 | 11 | C | ACAT1 | -0.002524 | 0.002437 | 0.300332 |
| 108013093 | rs10890818 | 11 | A | ACAT1 | 0.000111 | 0.002637 | 0.966494 |
| 108912668 | rs6533329 | 4 | A | HADH | -0.000421 | 0.002772 | 0.879387 |
| 108931551 | rs141066 | 4 | A | HADH | 0.001622 | 0.002114 | 0.443067 |
| 109535809 | rs1018782 | 12 | A | ACACB | -0.004697 | 0.00263 | 0.07411 |
| 109549579 | rs7978946 | 12 | C | ACACB | -0.000107 | 0.002371 | 0.96388 |
| 109592483 | rs11610260 | 12 | T | ACACB | -0.000281 | 0.002317 | 0.903603 |
| 109693348 | rs55960723 | 12 | G | ACACB | -0.001695 | 0.003203 | 0.596814 |
| 109740442 | rs11066182 | 12 | G | ACACB | -0.00231 | 0.002065 | 0.263215 |
| 109740593 | rs11831762 | 12 | C | ACACB | 0.001113 | 0.003002 | 0.710836 |
| 110621820 | rs5030539 | 4 | A | PLA2G12A | -0.002858 | 0.001921 | 0.13668 |
| 110651146 | rs11555260 | 4 | C | PLA2G12A | 0.00183 | 0.001944 | 0.346521 |
| 117421595 | rs11807991 | 1 | G | PTGFRN | 0.000844 | 0.002349 | 0.719454 |
| 117487884 | rs10923173 | 1 | G | PTGFRN | -0.000515 | 0.001978 | 0.794711 |
| 117514334 | rs6687760 | 1 | A | PTGFRN | -0.00175 | 0.00265 | 0.509077 |
| 117540386 | rs2057592 | 1 | A | PTGFRN | -0.000254 | 0.002047 | 0.901295 |
| 117550327 | rs2806873 | 1 | C | PTGFRN | -2.70E-05 | 0.002358 | 0.990859 |
| 120097006 | rs7967558 | 12 | T | PRKAB1 | 0.001542 | 0.001982 | 0.436664 |
| 120105707 | rs6490265 | 12 | T | PRKAB1 | -0.001167 | 0.002521 | 0.64331 |
| 125139340 | rs35119072 | 9 | T | PTGS1 | -0.001137 | 0.002669 | 0.670035 |
| 125164936 | rs10513402 | 9 | T | PTGS1 | 0.002355 | 0.003162 | 0.456484 |
| 125180343 | rs2778618 | 9 | A | PTGS1 | -0.00142 | 0.002643 | 0.591052 |
| 130850583 | rs10987870 | 9 | T | PTGES2 | 0.00147 | 0.002819 | 0.602022 |
| 132523860 | rs10118377 | 9 | A | PTGES | 0.003233 | 0.002077 | 0.119583 |
| 132538919 | rs1017509 | 9 | T | PTGES | 0.003183 | 0.002708 | 0.239828 |
| 132552936 | rs11793199 | 9 | T | PTGES | 0.004132 | 0.002178 | 0.057843 |
| 139463264 | rs1011018 | 7 | G | TBXAS1 | 0.00225 | 0.002512 | 0.370273 |
| 139523858 | rs1990354 | 7 | A | TBXAS1 | -0.003048 | 0.001896 | 0.107959 |
| 139553641 | rs41706 | 7 | T | TBXAS1 | 0.00278 | 0.002099 | 0.185301 |
| 139596859 | rs2267691 | 7 | C | TBXAS1 | 0.00201 | 0.002905 | 0.488929 |
| 139609317 | rs1978180 | 7 | C | TBXAS1 | -0.003372 | 0.002779 | 0.224986 |
| 139618001 | rs2299891 | 7 | T | TBXAS1 | -0.000513 | 0.002031 | 0.800441 |
| 139640896 | rs4726473 | 7 | T | TBXAS1 | 0.003432 | 0.002123 | 0.105949 |
| 139687783 | rs2267703 | 7 | G | TBXAS1 | -0.003902 | 0.001973 | 0.047939 |
| 139695316 | rs2284212 | 7 | G | TBXAS1 | 0.002979 | 0.002074 | 0.150866 |
| 139741766 | rs10267006 | 7 | C | TBXAS1 | -0.000371 | 0.002414 | 0.877858 |
| 139761463 | rs7794528 | 7 | C | TBXAS1 | 0.000202 | 0.002208 | 0.927084 |
| 139763419 | rs6943771 | 7 | C | TBXAS1 | -0.001847 | 0.002973 | 0.53447 |
| 139822542 | rs908831 | 9 | C | PTGDS | -0.002384 | 0.001863 | 0.200802 |
| 146610792 | rs4397700 | 1 | C | PRKAB2 | 0.006252 | 0.002992 | 0.036677 |
| 146641686 | rs3766522 | 1 | A | PRKAB2 | -0.003339 | 0.002141 | 0.118846 |
| 150369314 | rs57652693 | 5 | G | GPX3 | -0.001963 | 0.002598 | 0.44998 |
| 150384959 | rs2054440 | 5 | A | GPX3 | 0.004118 | 0.001818 | 0.023539 |
| 150400587 | rs870407 | 5 | A | GPX3 | -0.001864 | 0.002773 | 0.501492 |
| 150404311 | rs8177433 | 5 | C | GPX3 | -0.000753 | 0.002453 | 0.758927 |
| 150428584 | rs4958878 | 5 | A | GPX3 | 0.002205 | 0.003059 | 0.470973 |
| 150428871 | rs6862024 | 5 | G | GPX3 | -0.000708 | 0.002007 | 0.724353 |
| 150455672 | rs3792784 | 5 | A | GPX3 | -0.003201 | 0.003012 | 0.287862 |
| 175411953 | rs9312555 | 4 | A | HPGD | -0.002926 | 0.002634 | 0.266747 |
| 175442822 | rs1365613 | 4 | T | HPGD | 0.002779 | 0.0025 | 0.266322 |
| 175444281 | rs17553108 | 4 | G | HPGD | -0.003471 | 0.002794 | 0.214039 |
| 175463488 | rs2256673 | 4 | T | HPGD | 0.003167 | 0.002529 | 0.210531 |
| 175463555 | rs2256669 | 4 | C | HPGD | 0.000415 | 0.002294 | 0.856476 |
| 175475048 | rs17060632 | 4 | A | HPGD | -0.000425 | 0.002695 | 0.874687 |
| 179177116 | rs55827522 | 5 | C | LTC4S | 0.002019 | 0.002066 | 0.328502 |
| 179250392 | rs10464093 | 5 | G | LTC4S | 0.001228 | 0.001982 | 0.535419 |
| 186612725 | rs10911899 | 1 | T | PTGS2 | 0.000582 | 0.002963 | 0.844208 |
| 186648197 | rs5277 | 1 | C | PTGS2 | 0.004065 | 0.002479 | 0.101022 |
| 186649221 | rs2745557 | 1 | A | PTGS2 | -0.003705 | 0.002535 | 0.14383 |
| 186650751 | rs689466 | 1 | T | PTGS2 | -0.002256 | 0.002507 | 0.368207 |
| 186650846 | rs689465 | 1 | T | PTGS2 | 3.80E-05 | 0.0028 | 0.989154 |
| 186774893 | rs12757858 | 1 | T | PLA2G4A | -0.002026 | 0.00255 | 0.426865 |
| 186817928 | rs1473676 | 1 | C | PLA2G4A | 0.002644 | 0.00227 | 0.244071 |
| 186870263 | rs6695515 | 1 | G | PLA2G4A | 0.005182 | 0.002471 | 0.035991 |
| 186946638 | rs2891262 | 1 | C | PLA2G4A | 8.40E-05 | 0.002159 | 0.96901 |
| 186957215 | rs1555204 | 1 | C | PLA2G4A | 0.000683 | 0.002376 | 0.773846 |
| 186968711 | rs12139055 | 1 | A | PLA2G4A | 0.00089 | 0.002371 | 0.707479 |
| 186982931 | rs10911985 | 1 | G | PLA2G4A | -1.30E-05 | 0.001888 | 0.994526 |
| 219647487 | rs4674338 | 2 | G | PRKAG3 | -0.001102 | 0.001959 | 0.573722 |
| 219713015 | rs11691497 | 2 | C | PRKAG3 | 0.001327 | 0.002479 | 0.592318 |
