# Supplementary material for: Circulating n-3 fatty acids and trans-fatty acids, PLA2G2A gene variation and sudden cardiac arrest
Source: J Nutr Sci. 2016 Mar 1;5:e12. doi: 10.1017/jns.2016.2 (PMC4791519; doi:10.1017/jns.2016.2)
Supplement: Supplementary file 1 [file S2048679016000021sup.zip › S2048679016000021sup007.docx]

Supplementary table 7: Association of rs4654990 (C allele) with levels of fatty acids in 1318 SCA cases in VF

| **Fatty acid** | **beta** | **se** | **p-value** |
| --- | --- | --- | --- |
| DHA | -0.126 | 0.040 | 0.002 |
| EPA | -0.025 | 0.009 | 0.007 |
| Trans-18:1 | 0.063 | 0.024 | 0.007 |
| Trans-18:2 | 0.004 | 0.002 | 0.061 |
